# The late stage of COPI vesicle fission requires shorter forms of phosphatidic acid and diacylglycerol

**DOI:** 10.1038/s41467-019-11324-4

**Published:** 2019-07-30

**Authors:** Seung-Yeol Park, Jia-Shu Yang, Zhen Li, Pan Deng, Xiaohong Zhu, David Young, Maria Ericsson, Ruben L. H. Andringa, Adriaan J. Minnaard, Chunmei Zhu, Fei Sun, D. Branch Moody, Andrew J. Morris, Jun Fan, Victor W. Hsu

**Affiliations:** 1 Division of Rheumatology, Immunology and Allergy, Brigham and Women’s Hospital, Harvard Medical School, 60 Fenwood Road, Boston, MA 02115 USA; 2 Department of Medicine, Harvard Medical School, Boston, MA 02115 USA; 3 Department of Life Sciences, Pohang University of Science and Technology, Pohang, Gyeongbuk 37673 Republic of Korea; 4 Department of Materials Science and Engineering, City University of Hong Kong, Tat Chee Avenue, 999077 Hong Kong, China; 5 Division of Cardiovascular Medicine, Department of Medicine, University of Kentucky, 741S Limestone, Lexington, KY 40536 USA; 6 Lexington Veterans Affairs Medical Center, Lexington, KY 40536 USA; 7 Department of Cell Biology, Harvard Medical School, 220 Longwood Avenue, Boston, MA 02115 USA; 8 Stratingh Institute for Chemistry, University of Groningen, 9801 MX Groningen, The Netherlands; 9 National Key Laboratory of Biomacromolecules, CAS Center for Excellence in Biomacromolecules, Institute of Biophysics, Chinese Academy of Sciences, 15 Datun Road, Chaoyang District, 100101 Beijing, China; 10 University of Chinese Academy of Sciences, 100101 Beijing, China; 11 Center for Biological Imaging, Institute of Biophysics, Chinese Academy of Sciences, 100101 Beijing, China; 12 City University of Hong Kong, Shenzhen Research Institute, 518057 Shenzhen, China

**Keywords:** Golgi, Membrane fission

## Abstract

Studies on vesicle formation by the Coat Protein I (COPI) complex have contributed to a basic understanding of how vesicular transport is initiated. Phosphatidic acid (PA) and diacylglycerol (DAG) have been found previously to be required for the fission stage of COPI vesicle formation. Here, we find that PA with varying lipid geometry can all promote early fission, but only PA with shortened acyl chains promotes late fission. Moreover, diacylglycerol (DAG) acts after PA in late fission, with this role of DAG also requiring shorter acyl chains. Further highlighting the importance of the short-chain lipid geometry for late fission, we find that shorter forms of PA and DAG promote the vesiculation ability of COPI fission factors. These findings advance a general understanding of how lipid geometry contributes to membrane deformation for vesicle fission, and also how proteins and lipids coordinate their actions in driving this process.

## Abstract (web summary)

Phosphatidic acid and diacylglycerol are required for COPI vesicle fission. Here, the authors find that shorter forms of these lipids promote the late stage of COPI vesicle fission, further suggesting how lipid geometry contributes to membrane deformation in driving fission.

## Introduction

Vesicular transport is accomplished through a series of conserved steps, starting with the formation of vesicles from one intracellular compartment, followed by their targeting, docking, and then fusion with another compartment. How proteins deform membranes during vesicle formation is being elucidated in great detail^1–4^, but how lipids can also promote this process has been less understood.

A current view of how lipids can directly contribute to membrane deformation posits that the relative proportion between the head group and the acyl chains of phospholipids can produce either “cone” or “inverted-cone” geometry. Those that adopt inverted-cone geometry should induce positive membrane curvature, which is predicted to promote the budding stage of vesicle formation, while those with cone geometry should induce negative curvature, which is predicted to promote the fission stage^5,6^ (also summarized in Supplementary Fig. 1a). Notably, however, whereas biophysical and computational studies on model membranes have detailed how the shape and size of lipids can affect membrane deformation^7–9^, the complexity of native membranes, which contain a variety of lipids and proteins, has posed a formidable challenge in testing the role of a particular lipid geometry in a more physiologic setting.

Studies on Coat Protein I (COPI) complex highlight the importance of examining vesicle formation in the context of native membranes. Early studies identified a multimeric complex, known as coatomer^10^, to constitute the core components of the COPI complex, and ADP-ribosylation factor 1 (ARF1) as the small GTPase that regulates the recruitment of coatomer from the cytosol to Golgi membrane^11^. However, because native membranes contain peripheral membrane proteins that are recruited from the cytosol, we have subsequently used more stringently washed Golgi membrane to identify additional cytosolic proteins needed for COPI vesicle formation. These include a GTPase-activating protein (GAP) that acts on ARF1, known as ARFGAP1^12,13^, and Brefeldin-A ADP-ribosylated substrate (BARS)^14^.

The use of Golgi membrane has also led us to identify a key lipid needed for COPI vesicle formation. Initially, we found that phosphatidic acid (PA) cooperates with BARS in driving the fission stage^15^. Subsequently, how PA acts in this process has been revealed to be more complex. Whereas PA generated by lysophosphatidic acid acyltransferase type gamma (LPAAT−γ) promotes the early stage of COPI vesicle fission, PA generated by phospholipase D type 2 (PLD2) promotes the late stage^16^ (also summarized in Supplementary Fig. 1b). In addition, others have found that diacylglycerol (DAG) acts in COPI vesicle fission^17,18^. Thus how PA acts in COPI vesicle fission needs to be better understood, and its relation to DAG in this process remains to be elucidated.

In the current study, we find that PA with varying lipid geometry can all promote the early stage of COPI vesicle fission, but only PA with shorter acyl chains promotes late fission. We then find that DAG acts after PA in late fission, and this role of DAG also requires shorter acyl chains. These findings elucidate the mechanistic relationship between PA and DAG in COPI vesicle fission and also highlight the importance of the short-chain lipid geometry in this process. We also find that the shorter lipids promote the fission ability of ARF1, ARFGAP1, and BARS, thus advancing a general understanding of how lipids and proteins coordinate their actions in driving vesicle fission.

## Results

### PA of varying lipid geometry can all promote early fission

Led by the consideration that PA is defined by its polar head group, we explored whether differences in the acyl chains in PA could explain how it acts in complex ways during COPI vesicle fission. A vesicle reconstitution system, which involves the incubation of Golgi membrane with purified protein factors, has enabled the mechanistic details of COPI vesicle formation to be dissected out^12–16,19^. Using this approach previously, we had depleted PLD2 from Golgi membrane to inhibit COPI vesicle fission and then added PA to overcome this inhibition in confirming that PA generated by PLD2 activity is needed for COPI vesicle fission^15^. Notably, this rescue approach suggested a way of systematically interrogating how differences in the acyl chains of PA could affect its role in COPI vesicle fission.

**Fig. 1:**
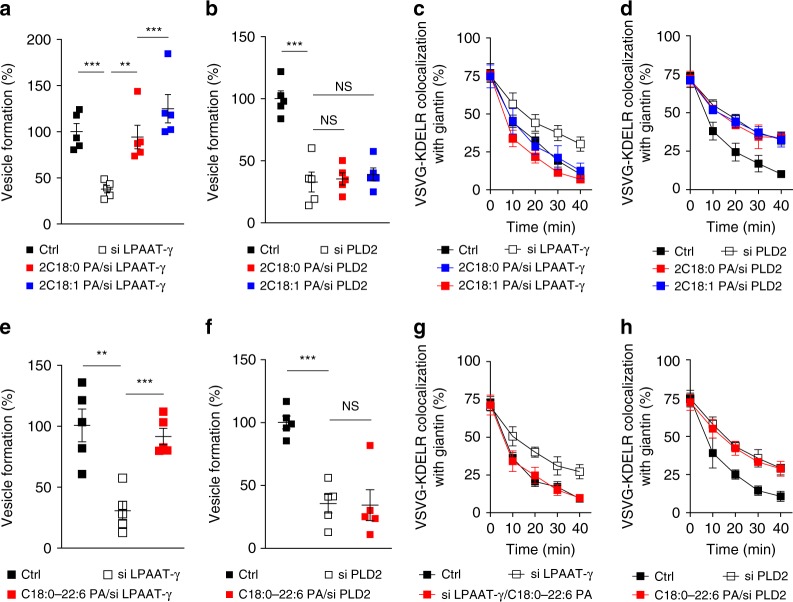
Phosphatidic acid (PA) in early Coat Protein I (COPI) vesicle fission. Quantitative data are shown as mean ± s.e.m. Significance was tested using the two-tailed Student’s *t* test, **P < 0.01, ***P < 0.0001, NS (non-significant) *P* > 0.05. **a** Golgi membrane with reduced lysophosphatidic acid acyltransferase type gamma (LPAAT−γ) level was used for the COPI vesicle reconstitution system. Rescue used PA forms as indicated; *n* = 5 independent experiments. **b** Golgi membrane with reduced phospholipase D type 2 (PLD2) level was used for the COPI vesicle reconstitution system. Rescue used PA forms as indicated; *n* = 5 independent experiments. **c** HeLa cells were treated with small interfering RNA (siRNA) against *LPAAT−γ*. Rescue used PA forms as indicated. COPI transport in cells was tracked by examining the quantitative colocalization of a COPI-dependent cargo protein (VSVG-KDELR) with Golgi marker (giantin) at different time points as indicated, *n* = 4 independent experiments. **d** HeLa cells were treated with siRNA against *PLD2*. Rescue used PA forms as indicated. COPI transport in cells was tracked by examining the quantitative colocalization of a COPI-dependent cargo protein (VSVG-KDELR) with Golgi marker (giantin) at different time points as indicated, *n* = 4 independent experiments. **e** Golgi membrane with reduced LPAAT−γ level was used for the COPI vesicle reconstitution system. Rescue used PA forms as indicated; *n* = 5 independent experiments. **f** Golgi membrane with reduced PLD2 level was used for the COPI vesicle reconstitution system. Rescue used PA forms as indicated; *n* = 5 independent experiments. **g** HeLa cells were treated with siRNA against *LPAAT−γ*. Rescue used PA forms as indicated. COPI transport in cells was tracked by examining the quantitative colocalization of a COPI-dependent cargo protein (VSVG-KDELR) with Golgi marker (giantin) at different time points as indicated, *n* = 4 independent experiments. **h** HeLa cells were treated with siRNA against *PLD2*. Rescue used PA forms as indicated. COPI transport in cells was tracked by examining the quantitative colocalization of a COPI-dependent cargo protein (VSVG-KDELR) with Golgi marker (giantin) at different time points as indicated, *n* = 4 independent experiments. Source data are provided as a Source Data file

Initially, to inhibit early fission, we treated cells with small interfering RNA (siRNA) against *LPAAT−γ *and then collected Golgi membrane as previously described^16^. When this Golgi membrane was used in the COPI reconstitution system, we found that PA with saturated acyl chains (2C18:0, see also Supplementary Fig. 1c) rescued the inhibition in early fission (Fig. 1a). PA with single unsaturation in both acyl chains (2C18:1, with *cis* double bond at carbon position 9), which results in a more “cone” geometry (see Supplementary Fig. 1d), also rescued the inhibition (Fig. 1a).

We then examined how these forms of PA act in late fission. Cells were treated with siRNA against *PLD2*, and Golgi membrane was again collected from the treated cells. When this Golgi membrane was used in the COPI reconstitution system, we found that neither form of PA, having saturated chains (2C18:0) or single unsaturated chains (2C18:1), could rescue the inhibition in late fission (Fig. 1b). We also confirmed that the siRNA treatments were efficient, as assessed by the expression of a scrambled siRNA labeled with a fluorophore (Supplementary Fig. 1e). Immunoblotting also confirmed the efficient reduction in the level of LPAAT−γ (Supplementary Fig. 1f) and PLD2 (Supplementary Fig. 1g) upon siRNA treatment. Thus these initial results suggested that early fission is not particularly sensitive to changes in the “cone” versus “inverted-cone” geometry of PA, while late fission requires a form of PA that remains to be defined.

We also pursued cell-based studies to confirm the above findings. An in vivo transport assay has been established to track retrograde COPI transport, which follows the redistribution of a COPI-dependent cargo (VSVG-KDELR) from the Golgi to the endoplasmic reticulum (ER)^14–16,19^. We had previously pursued this cell-based approach to confirm key findings derived from the vesicle reconstitution approach^14–16,19^. As specific lipids can be delivered into cells through albumin-containing medium^20^, we treated cells with siRNA against *LPAAT−γ*, followed by incubation with this medium that contained different forms of PA. When PA with saturated acyl chains (2C18:0) was fed to cells, we observed rescue of COPI transport that had been inhibited by knocking down *LPAAT−γ* (Fig. 1c and Supplementary Fig. 2a). When PA with single unsaturations in both chains (2C18:1) was used, rescue of the inhibition was also observed (Fig. 1c and Supplementary Fig. 2a). Notably, however, neither form of PA could rescue the inhibition of COPI transport induced by siRNA against *PLD2* (Fig. 1d and Supplementary Fig. 2b). Thus these cell-based results were in complete agreement with those obtained from the vesicle reconstitution system.

Next, in search of a form of PA that could rescue the inhibition of late fission induced by targeting against PLD2, we noted that a previous study had found that a high degree of polyunsaturation in one acyl chain, resulting in this chain “curling up” toward the polar head group, promotes vesicle fission in clathrin-mediated endocytosis^20^. Thus we examined whether PA that adopts this type of configuration promotes COPI vesicle fission. When the reconstitution system was performed using Golgi membrane with reduced LPAAT−γ, we found that PA with polyunsaturation in one chain (C18:0/C22:6, see also Supplementary Fig. 2c) rescued the inhibition in early fission (Fig. 1e). However, when Golgi membrane with reduced PLD2 was used, we found that the polyunsaturated PA still could not rescue the inhibition in late fission (Fig. 1f). These results were again confirmed by cell-based studies, as the polyunsaturated PA could overcome the inhibition in COPI transport induced by treating cells with siRNA against *LPAAT−γ* (Fig. 1g and Supplementary Fig. 2d), but not the inhibition induced by treating cells with siRNA against *PLD2* (Fig. 1h and Supplementary Fig. 2e).

### Only PA with short acyl chains promotes late fission

**Fig. 2:**
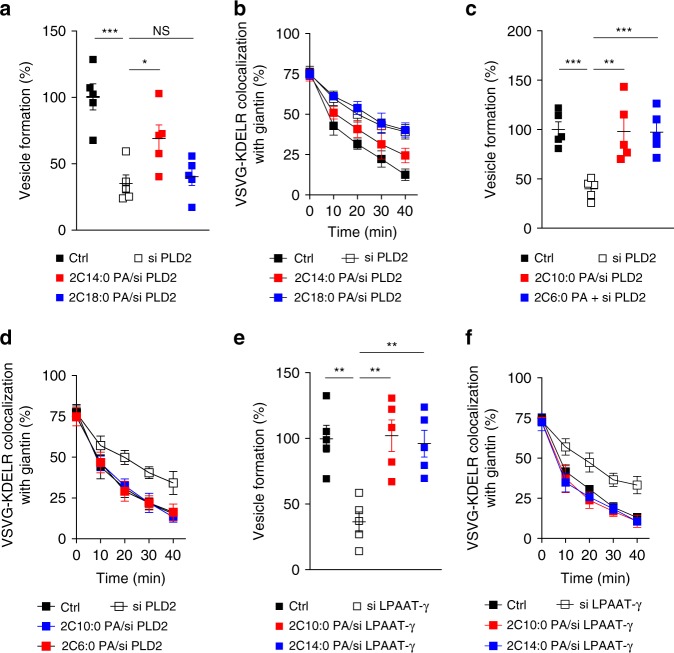
Phosphatidic acid (PA) in late Coat Protein I (COPI) vesicle fission. Quantitative data are shown as mean ± s.e.m. Significance was tested using the two-tailed Student’s *t* test, *P < 0.05, **P < 0.01, ***P < 0.0001, NS *P* > 0.05. **a** Golgi membrane with reduced phospholipase D type 2 (PLD2) level was used for the COPI vesicle reconstitution system, with rescue of vesicle formation using different forms of PA as indicated; *n* = 5 independent experiments. **b** HeLa cells were treated with small interfering RNA (siRNA) against *PLD2* to inhibit COPI transport, with rescue using different forms of PA as indicated. COPI transport in cells was tracked by examining the quantitative colocalization of a COPI-dependent cargo protein (VSVG-KDELR) with Golgi marker (giantin) at different time points as indicated, *n* = 4 independent experiments. **c** Golgi membrane with reduced PLD2 level was used for the COPI vesicle reconstitution system, with rescue of vesicle formation using PA having varying lengths of acyl chains as indicated; *n* = 5 independent experiments. **d** HeLa cells were treated with siRNA against *PLD2* to inhibit COPI transport, with rescue using PA having varying lengths of acyl chains as indicated. COPI transport in cells was tracked by examining the quantitative colocalization of a COPI-dependent cargo protein (VSVG-KDELR) with Golgi marker (giantin) at different time points as indicated, *n* = 4 independent experiments. **e** Golgi membrane with reduced lysophosphatidic acid acyltransferase type gamma (LPAAT−γ) level was used for the COPI vesicle reconstitution system, with rescue of vesicle formation using different forms of PA as indicated; *n* = 5 independent experiments. **f** HeLa cells were treated with siRNA against *LPAAT−γ* to inhibit COPI transport, with rescue using different forms of PA as indicated. COPI transport in cells was tracked by examining the quantitative colocalization of a COPI-dependent cargo protein (VSVG-KDELR) with Golgi marker (giantin) at different time points as indicated, *n* = 4 independent experiments. Source data are provided as a Source Data file

We next considered that, besides modifying the saturation status of the acyl chains, the other general way of modifying acyl chains would be to alter their length. In this regard, lipids with shorter acyl chains have been observed in biological membranes^21,22^, but their roles remain to be better understood. Thus we examined whether shortening the acyl chains in PA affects COPI vesicle fission. Performing the vesicle reconstitution system, we found that PA with shorter acyl chains (2C14:0) could indeed rescue the inhibition in late fission induced by targeting against PLD2 on Golgi membrane (Fig. 2a). This result was also confirmed by the COPI transport assay (Fig. 2b and Supplementary Fig. 3a).

Because the rescue using PA (2C14:0) was partial, we next examined whether further shortening the acyl chains would lead to a more complete rescue. Performing the vesicle reconstitution system, we confirmed that PA with even shorter acyl chains (2C10:0) was more efficient in rescuing the inhibition of late fission induced by depleting PLD2 on Golgi membrane (Fig. 2c). Further shortening the acyl chains of PA (2C6:0) did not result in further enhanced rescue of COPI vesicle formation (Fig. 2c), suggesting that optimal rescue effect had been reached when PA was reduced to C10 in length. These results were also confirmed by the COPI transport assay, as the shorter PA (2C10:0) completely restored COPI transport that had been inhibited by siRNA against *PLD2*, while the use of an even shorter form of PA (2C6:0) did not show further enhancement in this transport (Fig. 2d and Supplementary Fig. 3b).

We also examined whether shortening the acyl chains in PA affects its role in early fission. Performing the COPI reconstitution system, we found that altering the length of acyl chains in PA did not affect its ability to rescue the inhibition in early fission induced by targeting against LPAAT−γ on Golgi membrane (Fig. 2e). This result was also confirmed by the COPI transport assay (Fig. 2f and Supplementary Fig. 3c). Altogether, the above results revealed that late fission is sensitive to the length of acyl chains in PA, but early fission is not.

### Only short DAG promotes late fission

We then pursued another line of investigation that further supported the importance of shorter acyl chains in late fission. Besides PA, DAG has also been suggested to act in COPI vesicle fission^17,18^. However, because only cell-based studies had been performed^17,18^, uncertainty exists whether DAG could be acting indirectly to promote COPI vesicle fission. Thus we initially addressed this issue by performing the COPI reconstitution system.

**Fig. 3:**
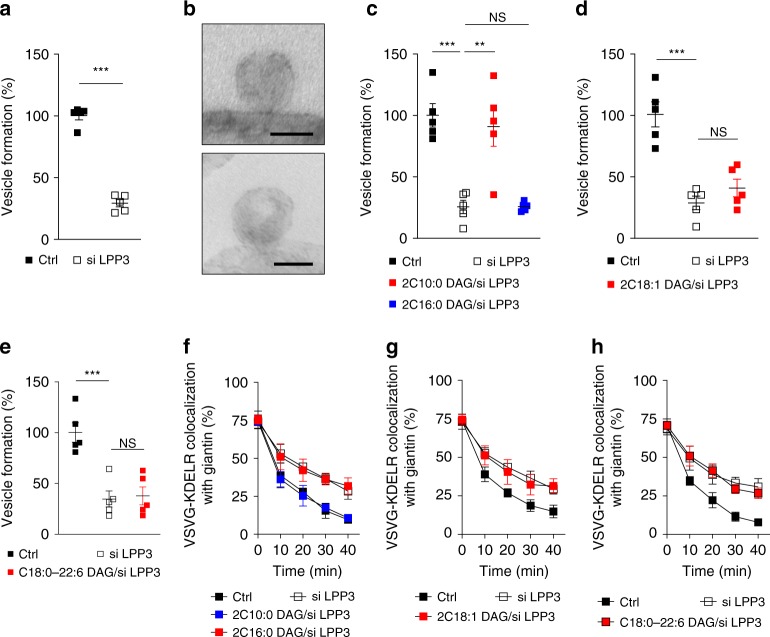
Diacylglycerol (DAG) in late Coat Protein I (COPI) vesicle fission. Quantitative data are shown as mean ± s.e.m. Significance was tested using the two-tailed Student’s *t* test, **P < 0.01, ***P < 0.0001, NS *P* > 0.05. **a** Golgi membrane with reduced lipid phosphate phosphatase type 3 (LPP3) level was used for the COPI vesicle reconstitution system; *n* = 5 independent experiments. **b** Golgi membrane was isolated from HeLa cells treated with small interfering RNA (siRNA) against *LPP3* and then used for incubation in the COPI vesicle reconstitution system. COPI buds on Golgi membrane were then detected by electron microscopy, with representative images shown, bar = 50 nm, *n* = 3 independent experiments. **c** Golgi membrane with reduced LPP3 level was used for the COPI vesicle reconstitution system, with rescue of vesicle formation using DAG having varying length of acyl chains as indicated; *n* = 5 independent experiments. **d** Golgi membrane with reduced LPP3 level was used for the COPI vesicle reconstitution system, with rescue of vesicle formation using DAG with single unsaturations; *n* = 5 independent experiments. **e** Golgi membrane with reduced LPP3 level was used for the COPI vesicle reconstitution system, with rescue of vesicle formation using a polyunsaturated DAG; *n* = 5 independent experiments. **f** HeLa cells were treated with siRNA against *LPP3* to inhibit COPI transport, with rescue using DAG having varying lengths of acyl chains as indicated. COPI transport in cells was tracked by examining the quantitative colocalization of a COPI-dependent cargo protein (VSVG-KDELR) with Golgi marker (giantin) at different time points as indicated, *n* = 4 independent experiments. **g** HeLa cells were treated with siRNA against *LPP3* to inhibit COPI transport, with rescue using DAG having single unsaturations. COPI transport in cells was tracked by examining the quantitative colocalization of a COPI-dependent cargo protein (VSVG-KDELR) with Golgi marker (giantin) at different time points as indicated, *n* = 4 independent experiments. **h** HeLa cells were treated with siRNA against *LPP3* to inhibit COPI transport, with rescue using a polyunsaturated DAG. COPI transport in cells was tracked by examining the quantitative colocalization of a COPI-dependent cargo protein (VSVG-KDELR) with Golgi marker (giantin) at different time points as indicated, *n* = 4 independent experiments. Source data are provided as a Source Data file

DAG that promotes COPI vesicle fission has been suggested to be generated from PA through the activity of lipid phosphate phosphatase type 3 (LPP3)^18^. Thus we treated cells with siRNA against *LPP3* and then isolated Golgi membrane for the reconstitution system. We first confirmed that LPP3 level was reduced (Supplementary Fig. 4a). We then found that COPI vesicle formation was inhibited by this treatment (Fig. 3a). Further characterizing this inhibition, we found by electron microscopy (EM) that Golgi membrane accumulated buds with severely constricted necks (Fig. 3b). Thus the results not only confirmed that DAG acts in COPI vesicle fission but also revealed that this role occurs in late fission.

Such a role for DAG suggested another way of testing the importance of acyl chain length in late fission. Pursuing the COPI reconstitution system, we found that short DAG (2C10:0) rescues the inhibition in late fission induced by depleting LPP3 level in Golgi membrane (Fig. 3c). In contrast, DAG of longer length (2C16:0) could not (Fig. 3c). We also examined how different unsaturated forms of DAG affects its role in late fission. DAG with single unsaturations in both chains (2C18:1) could not rescue the inhibition in late fission induced by depleting LPP3 in Golgi membrane (Fig. 3d). DAG with polyunsaturation in one chain (C18:0/C22:6) (Fig. 3e) also could not rescue this inhibition.

We also sought to confirm these findings by pursuing cell-based studies. Feeding cells with DAG of shorter acyl chains (2C10:0) rescued the inhibition in COPI transport induced by siRNA against *LPP3* (Fig. 3f and Supplementary Fig. 4b). In contrast, DAG with longer acyl chains (2C16:0) (Fig. 3f and Supplementary Fig. 4b), with single unsaturations in both chains (2C18:1) (Fig. 3g and Supplementary Fig. 4c) or with polyunsaturation in one chain (C18:0/C22:6) (Fig. 3h and Supplementary Fig. 4d), could not. Thus, similar to that seen above for the role of PA in late fission, the role of DAG in late fission is also selective for shortened acyl chains.

We also confirmed that the roles of short PA and DAG in late fission are distinct. Performing the COPI reconstitution system, we found that the inhibition of late fission induced by PLD2 depletion, which was rescued by short PA, could not be rescued by short DAG (Supplementary Fig. 5a). Moreover, inhibition of late fission induced by LPP3 depletion, which was rescued by short DAG, could not be rescued by short PA (Supplementary Fig. 5b). These results were also confirmed by cell-based studies. Whereas inhibition of COPI transport induced by PLD2 is rescued by short PA, this inhibition cannot be rescued by short DAG (Supplementary Fig. 5c, d). Similarly, whereas inhibition of COPI transport induced by LPP3 is rescued by short DAG, this inhibition cannot be rescued by short PA (Supplementary Fig. 5e, f). Further confirming the specificity by which PLD2 and LPP3 acts, we found that short phosphatidylcholine (PC; 2C10:0) could not rescue inhibition induced by targeting against *PLD2* or *LPP3* (Supplementary Fig. 5g). Altogether, when also considering that LPP3 activity converts PA to DAG, we concluded that late fission likely involves the sequential actions of short PA followed by short DAG.

### Membrane properties conducive for fission

**Fig. 4:**
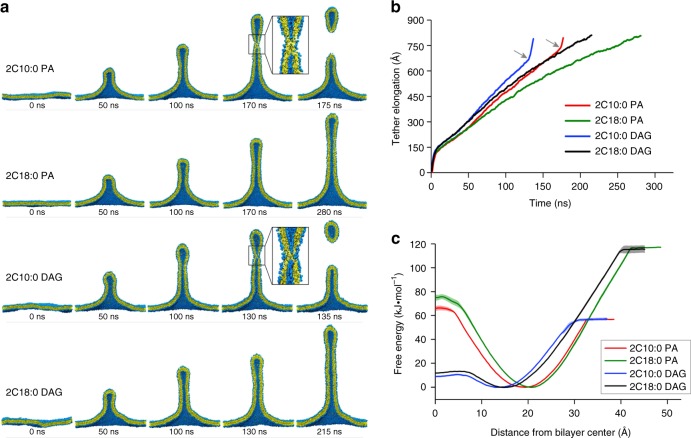
Coarse-grained molecular dynamics simulations. Membranes contain 70% dioleoyl-phosphatidylcholine and 30% of phosphatidic acid (PA) or diacylglycerol (DAG) in forms as indicated. **a** When force is exerted on membranes that contain shorter (2C10:0) PA or DAG, membrane tubulation occurs, followed by fission. However, when force is exerted on membranes that contain longer (2C18:0) PA or DAG, membrane tubulation occurs, but no fission is observed. Cross-sections of tubulated membranes are shown. Water and ions are not shown for clarity. Fission events are highlighted by inset, which shows the fusion of the inner layer of the tubulated membrane bilayer. Replica of four was performed, with a representative result shown. **b** Membranes containing shorter PA/DAG possess higher elongation rate and undergo a sudden inflection (indicated by gray arrows and corresponding to membrane fission) when the elongation distance reaches ~700 Å. **c** Potential of mean force (PMF) profiles for different forms of PA and DAG. PMF was set to zero at the equilibrium position (free energy minimum) of a lipid in the membrane bilayer, which is approximately 15–20 Å from the center of the bilayer. For a lipid moving away from the bilayer center (toward 40 Å), the free energy barrier is lower for shorter PA/DAG. For a lipid moving toward the bilayer center (toward 0 Å), the free energy barrier is lower for DAG

To gain further insight how these short lipids could promote late fission, we next pursued coarse-grained (CG) molecular dynamics (MD) simulation studies, an approach that has been used previously to predict how lipid properties could affect membrane fission^20,23^. We mimicked membrane deformation by exerting a pulling force onto model membranes, as previously described^20,23^. Moreover, we modeled membranes to contain 70% dioleoyl-phosphatidylcholine (DOPC, 2C18:1) and 30% of a lipid of interest, PA or DAG in their shorter (2C10:0) or longer (2C18:0) form. Simulations revealed that the pulling force induces the tubulation of all membranes, but notably, only membranes that contain short PA or DAG underwent fission (Fig. 4a). Further scrutiny revealed that tubular membranes containing shorter PA or DAG elongated at a higher rate and then underwent a sudden inflection point when the tubular extension reached near 700 Å (Fig. 4b), suggesting that a membrane fission event had occurred. We also analyzed membrane properties and found that the shorter PA and DAG reduced membrane thickness and rigidity and increased lipid lateral diffusion (Table 1), properties that are all conducive for membrane deformation.

**Table 1:** Effect of shorter acyl chains on membrane properties

	2C10:0 PA	2C18:0 PA	2C10:0 DAG	2C18:0 DAG
Thickness (Å)	36.33 ± 0.05	39.15 ± 0.06	37.58 ± 0.08	40.69 ± 0.10
*D*_L_ (μm^2^ s^−1^)	67.44 ± 1.34	56.49 ± 0.71	86.84 ± 1.71	66.46 ± 1.90
*K*_C_ (*k*_B_T)	25.15 ± 2.15	30.61 ± 6.92	11.19 ± 1.70	11.29 ± 1.34

*D*_*L*_ lipid lateral diffusion coefficient, *DAG* diacylglycerol, *PA* phosphatidic acid, *K*_*C*_ bending rigidity

We next considered that membrane fission involves the progressive constriction of a bud neck, which eventually results in the apposition of the inner layer of the neck membrane to promote membrane fusion, a process that is predicted to be facilitated by lipid tails becoming solvent exposed^24^. Calculating the energy profile of PA/DAG for this process, we found that it is thermodynamically more favorable for shorter PA and DAG to move perpendicularly out of the membrane toward the solvent (Fig. 4c). Another behavior of lipids that has been predicted to promote fission is lipid flipping, as this process promotes the hemi-fusion stage of fission^25–27^. Calculating the energy profile of PA/DAG for this process, we found that it is thermodynamically more favorable for DAG than PA to move toward the inner core of the membrane bilayer (Fig. 4c). Thus these results provided further insights into how short PA/DAG promote late fission.

### Detection of short PC on Golgi membrane

We next noted that lipidomics studies thus far have not detected phospholipids with short acyl chains (in the range of 10-carbon length). Instead, lipids with mixed lengths have been observed, with the shortest having one acyl chain being C10 and the other being C16 in length^21,22^. Thus we explored the possibility that such mixed forms of PA and DAG could be the physiologic lipids that promote late fission. However, we found that PA (either C16:0/C10:0 or C10:0/C16:0, Supplementary Fig. 6a) could not rescue the block in COPI vesicle fission induced by targeting against PLD2 (Supplementary Fig. 6b), and DAG (either C16:0/C10:0 or C10:0/C16:0, Supplementary Fig. 6c) also could not rescue the block induced by targeting against LPP3 (Supplementary Fig. 6d).

**Fig. 5:**
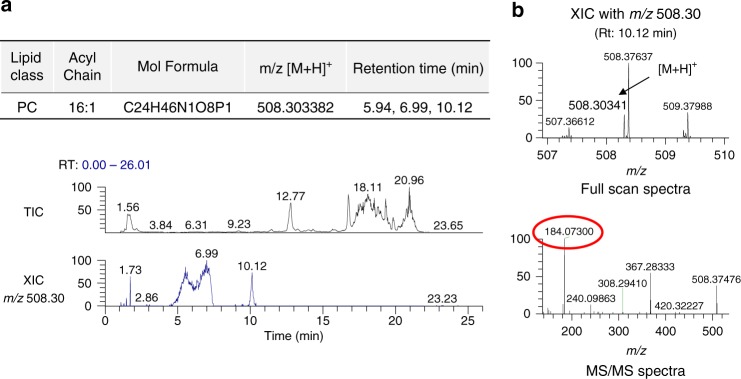
Lipid analysis of Golgi membrane. **a** Representative liquid chromatography-mass spectrometric chromatograms of the Golgi lipid extract. Shown is the total ion chromatogram and the extracted ion chromatogram (XIC) of phosphatidylcholine (PC) (16:1) with *m*/*z* 508.30. Note that multiple peaks were detected in the XIC channel indicating that there are PC isomers with different acyl chain length. Peaks detected at 5.94, 6.99, and 10.12 min for *m*/*z* 508.30 are consistent with PC having different acyl chain length combinations of 10:0/6:1, 8:0/8:1, or 10:1/6:0. **b** Representative precursor and product ion spectra of XIC with *m*/*z* 508.30. The precursor ions are assigned with mass error <3 ppm. Characteristic fragment at *m*/*z* 184.07 (representing choline phosphate) further confirms the identity of PC

We then considered that lipidomics studies in general are not tailored to detect short lipids. Thus, guided by short lipids as standards, we adjusted the lipid extraction procedure and also lipid elution during chromatography, which then allowed us to detect PC with short acyl chains on the Golgi membrane by mass spectrometry (MS). Specifically, whereas previous lipidomics studies had detected PC with combined chain length down to 26 carbons (e.g., C10:0/C16:0)^21,22^, we detected PC with combined chain length down to 16 carbons (Fig. 5a). Product ion spectra of this short PC were consistent with neutral losses of fatty acid carboxylate ions corresponding to acyl chains of C8:0/C8:1, C6:0/C10:1, or C6:1/C10:0 (Fig. 5b). Thus, as PLD2 converts PC to PA and LPP3 converts PA to DAG, the detection of short PC suggested how short PA and DAG could be generated on Golgi membrane for COPI vesicle fission.

In contrast to the detection of short PC, we were unable to detect short PA and DAG on Golgi membrane. As PA and DAG are far less abundant than PC, a likely explanation was that short PA and DAG, being even more rare than short PC, were below the detection limit of our analysis. Consistent with this explanation, we found that feeding cells with short PA allowed us to detect the fed PA on Golgi membrane (Supplementary Fig. 7a, b). Notably, we also detected a minor level of short DAG on this fed Golgi membrane (Supplementary Fig. 7a, b), which supported our prediction above that the sequential actions of PLD2 and LPP3 would allow short PC to become short PA and then short DAG. Further notable, we found that Golgi membrane isolated from cells fed with short PA showed even higher level of short DAG when incubated in the COPI reconstitution system (Supplementary Fig. 7c).

### Shorter lipids promote the fission ability of COPI factors

We next considered that ARF1, ARFGAP1, and BARS have all been implicated to act in COPI vesicle fission^12,14,28^, but how lipid geometry could modulate the roles of these protein factors has not been explored. Furthermore, although the use of liposomes has become a standard approach of assessing the intrinsic ability of proteins to deform membrane^29–32^, the COPI fission factors had only been documented to induce liposome tubulation^15,33^, rather than vesiculation as would be expected of fission factors. Thus we next explore the intriguing possibility that short forms of PA and DAG could promote the vesiculation ability of these fission factors.

**Fig. 6:**
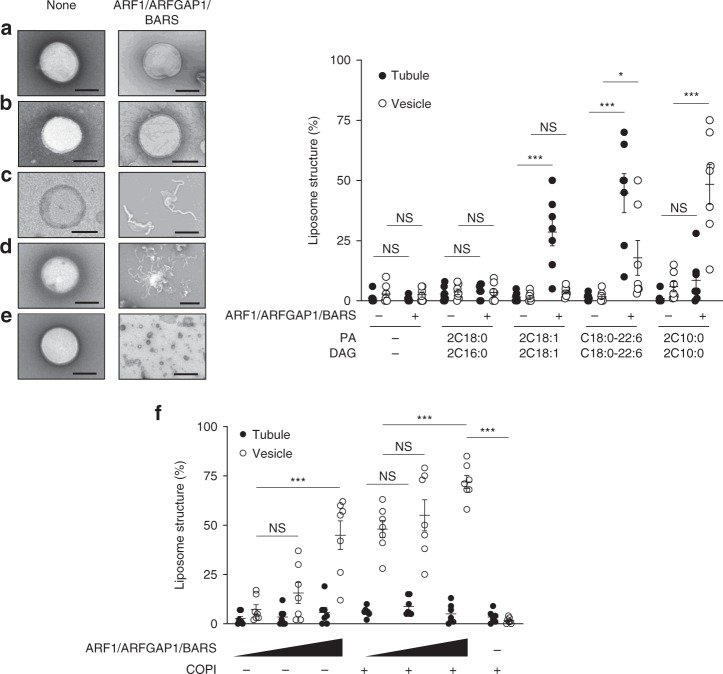
Reconstituting vesiculation with liposomes. Quantitative data are shown as mean ± s.e.m. Significance was tested using the two-tailed Student’s *t* test, *P < 0.05, ***P < 0.0001, NS *P* > 0.05. Golgi-like liposomes were generated with additional lipids incorporated as follows: **a** none, **b** phosphatidic acid (PA) (2C18:0) and diacylglycerol (DAG) (2C16:0), **c** PA (2C18:1) and DAG (2C18:1), **d** PA (C18:0/C22:6) and DAG (C18:0/C22:6), and **e** PA (2C10:0) and DAG (2C10:0). Liposomes were then incubated with Coat Protein I (COPI) fission factors as indicated, followed by electron microscopic (EM) examination. Representative images are shown on left, bar = 250 nm. The degrees of liposome tubulation and vesiculation are quantified on right; *n* = 7 independent experiments. **f** Golgi-like liposomes with PA (2C10:0) and DAG (2C10:0) were incubated with increasing levels of COPI fission factors (ARF1, ARFGAP1, and BARS) and either with or without coatomer, followed by EM examination to assess the degree of liposome tubulation and vesiculation; *n* = 3 independent experiments. Source data are provided as a Source Data file

Previous studies had mimicked the lipid composition of the Golgi membrane by generating liposomes that contain 50% DOPC, 10% phosphatidylethanolamine (PE), 7% phosphatidylserine (PS), 6% phosphatidylinositol (PI), 17% cholesterol, and 8% sphingomyelin (SM)^15,34^. Thus we generated these liposomes and also incorporated PA and DAG in their different forms at 1% each. Initially, we confirmed that, without the key lipids (PA and DAG), the Golgi-like liposomes could not support a significant level of membrane deformation by the COPI fission factors (Fig. 6a). Liposomes that contained the fully saturated forms (2C18:0) of these key lipids also could not support a significant level of liposome deformation (Fig. 6b). In contrast, liposomes that contained other geometries of the key lipids promoted the deformation of liposomes by the fission factors (Fig. 6c, d), and notably, liposomes that contain the short forms of key lipid had the best ability in supporting membrane vesiculation by the COPI fission factors (Fig. 6e). Moreover, the presence of coatomer further enhanced the cooperation between these fission factors and short PA/DAG in promoting liposome vesiculation (Fig. 6f).

To further define which specific short lipid best promotes the vesiculation ability of a particular fission factor, we next pursued a comprehensive screen. To facilitate such a screen, we generated simplified liposomes, containing 70% DOPC and 30% of PA or DAG (in their various forms). We used a relatively high concentration of PA/DAG, reasoning that the local concentration of these lipids may be higher than its overall concentration on Golgi membrane. Consistent with this possibility, we found that both PLD2 and LPP3 interacts with BARS (Supplementary Fig. 8a), which we had found previously to be concentrated at the neck of COPI buds^14^. We further noted that previous studies had shown that ARF1 must be loaded with GTP in order to bind membrane for deformation^33,35,36^. As confirmation, we found that ARF1 must also be loaded with GTP in order to bind our simplified liposomes (Supplementary Fig. 8b).

We also considered that, because the different forms of PA/DAG used for the simplified liposomes have shape/length that differ from that of DOPC, such mismatch might prevent the formation of membrane bilayers. However, multiple lines of evidence ruled against this possibility. First, simulation studies predicted that the simplified liposomes with 30% of PA or DAG (in their various forms) should form membrane bilayers (Fig. 4a). Second, performing a liposome leakage assay, which involved the use of a calcium-sensitive fluorophore to monitor calcium escaping from inside of liposomes as previously described^37^, we found that the simplified liposomes with different forms of DAG are functionally intact (Supplementary Fig. 9a). Third, pursuing high-resolution cryo-EM, we directly visualized the membrane bilayer of these simplified liposomes (Supplementary Fig. 9b).

**Fig. 7:**
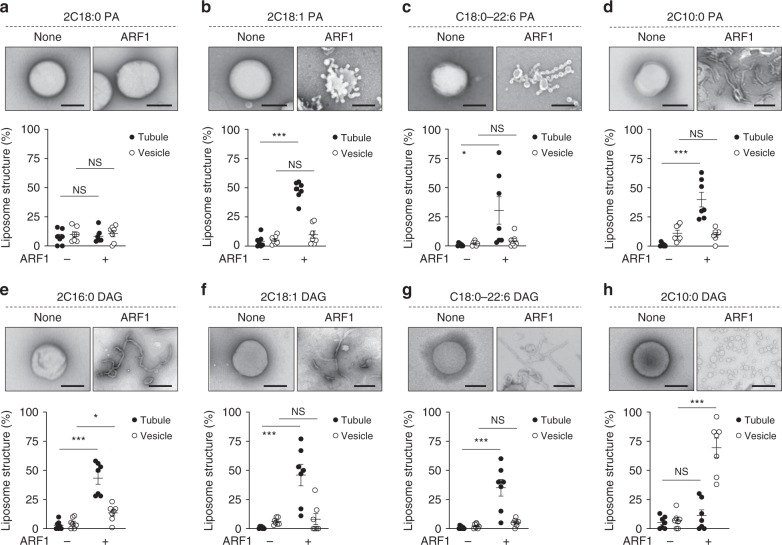
Short diacylglycerol (DAG) promotes vesiculation by ADP-ribosylation factor 1 (ARF1). Quantitative data are shown as mean ± s.e.m. Significance was tested using the two-tailed Student’s *t* test, *P < 0.05, ***P < 0.0001, NS *P* > 0.05. **a**–**d** Liposomes with different forms of phosphatidic acid as indicated were incubated with recombinant ARF1 and then examined by electron microscopy (EM). Representative images are shown above, bar = 250 nm. Quantitation of liposome tubulation and vesiculation is shown below; *n* = 4 independent experiments. **e**–**h** Liposomes with different forms of DAG as indicated were incubated with recombinant ARF1 and then examined by EM. Representative images are shown above, bar = 250 nm. Quantitation of liposome tubulation and vesiculation is shown below; *n* = 4 independent experiments. Source data are provided as a Source Data file

We then proceeded to examine how the vesiculation ability of different COPI fission factor could be affected by different forms of PA and DAG. Examining ARF1 initially, we found that the saturated form of PA (2C18:0) had little ability to promote either liposome tubulation or vesiculation by ARF1 (Fig. 7a). In comparison, liposomes that contain PA with single unsaturation in acyl chains (2C18:1) promoted the ability of ARF1 to induce short tubules (Fig. 7b). Liposomes that contain PA with polyunsaturation in one chain (C18:0/C22:6) allowed ARF1 to induce more extensive tubulation (Fig. 7c). These tubules often contained constrictions, suggesting that polyunsaturation was more potent in promoting the fission ability of ARF1. PA with shorter acyl chains (2C10:0) also promoted liposome tubulation by ARF1 (Fig. 7d). Thus multiple forms of PA could promote liposome tubulation to varying degree by ARF1. However, none could promote liposome vesiculation.

We next examined how the different geometries of DAG affect the fission ability of ARF1. DAG in its saturated form (2C16:0) promoted liposome tubulation by ARF1 (Fig. 7e). Single unsaturation of both acyl chains (2C18:1) (Fig. 7f) or polyunsaturation of an acyl chain (C18:0/C22:6) (Fig. 7g) also promoted liposome tubulation by ARF1. Remarkably, however, short DAG (2C10:0) enabled ARF1 to induce liposome vesiculation to virtual completion (Fig. 7h). Thus the results identified DAG with short acyl chains as the only lipid form able to induce liposome vesiculation by ARF1.

**Fig. 8:**
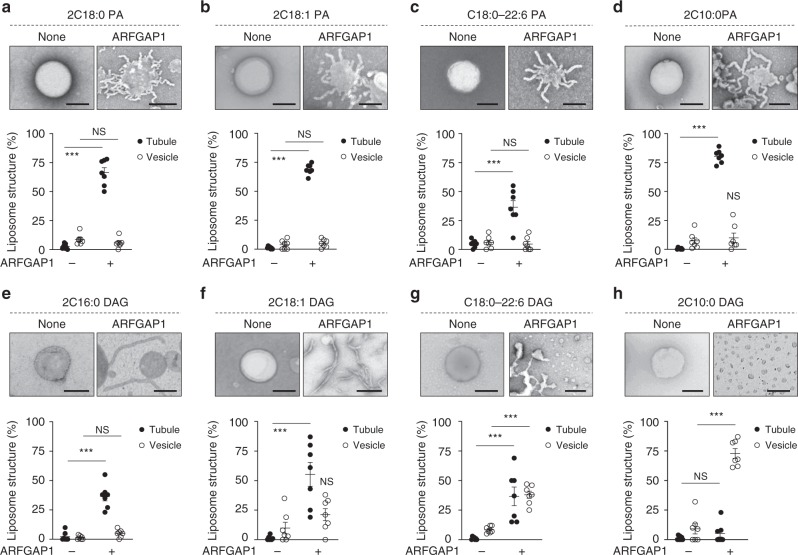
Short diacylglycerol (DAG) promotes vesiculation by ARFGAP1. Quantitative data are shown as mean ± s.e.m. Significance was tested using the two-tailed Student’s *t* test, ***P < 0.0001, NS *P* > 0.05. **a**–**d** Liposomes with different forms of phosphatidic acid as indicated were incubated with recombinant ARFGAP1 and then examined by electron microscopy (EM). Representative images are shown above, bar = 250 nm. Quantitation of liposome tubulation and vesiculation is shown below; *n* = 4 independent experiments. **e**–**h** Liposomes with different forms of DAG as indicated were incubated with recombinant ARFGAP1 and then examined by EM. Representative images are shown above, bar = 250 nm. Quantitation of liposome tubulation and vesiculation is shown below; *n* = 4 independent experiments. Source data are provided as a Source Data file

We then examined ARFGAP1. The saturated form of PA (2C18:0) enabled ARFGAP1 to induce liposome tubulation (Fig. 8a). Other forms of PA also promoted liposome tubulation by ARFGAP1. These included PA with single unsaturation in both acyl chains (2C18:1) (Fig. 8b), PA with polyunsaturation in one chain (C18:0/C22:6) (Fig. 8c), and PA with shorter acyl chains (Fig. 8d). With respect to DAG, we found that fully saturated (2C16:0) and single unsaturation in both chains (2C18:1) promoted liposome tubulation by ARFGAP1 (Fig. 8e, f). DAG with polyunsaturation in one chain (C18:0/C22:6) also promoted liposome tubulation by ARFGAP1, as well as some degree of vesiculation (Fig. 8g). Notably though, similar to that seen for ARF1, short DAG was also the best in promoting liposome vesiculation by ARFGAP1 (Fig. 8h).

**Fig. 9:**
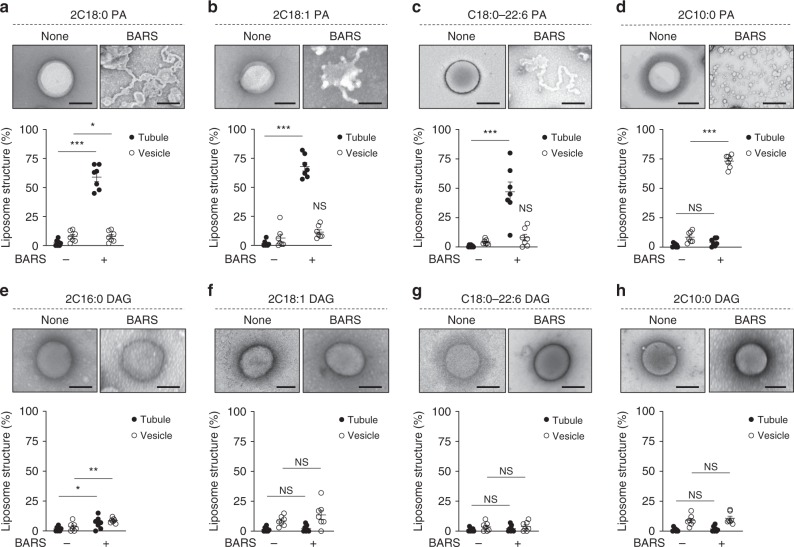
Short phosphatidic acid (PA) promotes vesiculation by Brefeldin-A ADP-ribosylated substrate (BARS). Quantitative data are shown as mean ± s.e.m. Significance was tested using the two-tailed Student’s *t* test, *P < 0.05, **P < 0.01, ***P < 0.0001, NS *P* > 0.05. **a**–**d** Liposomes with different forms of PA as indicated were incubated with recombinant BARS and then examined by electron microscopy (EM). Representative images are shown above, bar = 250 nm. Quantitation of liposome tubulation and vesiculation is shown below; *n* = 4 independent experiments. **e**–**h** Liposomes with different forms of diacylglycerol as indicated were incubated with recombinant BARS and then examined by EM. Representative images are shown above, bar = 250 nm. Quantitation of liposome tubulation and vesiculation is shown below; *n* = 4 independent experiments. Source data are provided as a Source Data file

Finally, examining BARS, we found that PA in its saturated form (2C18:0) (Fig. 9a), with single unsaturation in acyl chains (2C18:1) (Fig. 9b) or with polyunsaturation in one chain (C18:0/C22:6) (Fig. 9c), could all support liposome tubulation by BARS. However, when BARS was incubated with liposomes that contained short PA (2C10:0), we observed liposome vesiculation to virtual completion (Fig. 9d). In contrast, DAG in its various forms show little ability to promote liposome deformation by BARS (Fig. 9e–h). Thus, whereas short DAG best promotes the fission abilities of ARF1 and ARFGAP1, short PA best promotes the fission ability of BARS.

## Discussion

Examining how lipid geometry acts in COPI vesicle fission, we have found that PA of various lipid geometry can all promote the early stage of COPI vesicle fission. These include PA with acyl chains that are fully saturated, unsaturated to varying extent, or having shorter length. In contrast, late fission exhibits remarkable selectivity, as only PA with both acyl chains being shortened promotes this process. We have also clarified how DAG acts in COPI vesicle fission, pinpointing that it promotes specifically late fission, with this role also requiring both acyl chains being shortened. As this role of DAG requires its generation from LPP3 activity, which converts PA to DAG, the collective considerations lead us to conclude that late fission is accomplished through the sequential actions of short PA followed by short DAG.

We have found previously that BARS plays a key role in COPI vesicle fission, and this role requires BARS binding to PA^14,15^. In the current study, we have further clarified that PA of different forms can all promote early fission. Thus BARS, rather than the lipid geometry of PA, likely plays the dominant role in promoting membrane bending for the early stage of COPI vesicle fission. In the case of late fission, however, because we have found this process requires shortened acyl chains of PA and DAG, we have also pursued simulation studies to gain further insight into how these short lipids promote late fission. Examining membrane properties, we have found that these short lipids promote properties of the membrane that are conducive to its deformation. These include decreasing membrane thickness and bending rigidity, as well as increasing the lateral diffusion of lipids.

Besides these effects that are predicted to promote membrane deformation, we have also sought insight into how short PA and DAG promotes in particular the fission stage of vesicle formation. This process involves the progressive constriction of the bud neck, which ultimately results in the inner layer of the constricting neck membrane undergoing fusion, a process that has been predicted to be promoted by the solvent exposure of lipid tails^23^. We have calculated energy profiles for the movement of PA and DAG out of the membrane bilayer and have found that it is energetically more favorable for shorter acyl chains to become solvent exposed. Another process that has been predicted to be involved in membrane fission is lipid flipping, which has been predicted to facilitate the hemi-fusion stage of fission^8,27^. Calculating the energy profile for this process, we have found that it is energetically more favorable for DAG than PA to undergo lipid flipping. Notably, these predictions are also consistent with our experimental results that have placed short DAG to act later than short PA for COPI vesicle fission.

On a broader level, we have also gained insight into how key proteins and lipids coordinate their actions in promoting COPI vesicle fission. Examining the different protein factors that have been implicated to participate in this process, we find that short PA best promotes the vesiculation ability of BARS, while short DAG best promotes the vesiculation ability of ARF1 and ARFGAP1. We further note that, although the use of liposomes has revealed short PA and DAG to be best in promoting the vesiculation ability of COPI protein factors, polyunsaturated forms of these lipids also show some capability. In contrast, this type of lipid geometry does not exhibit a similar capability when assessed by the COPI transport assay or by the vesicle reconstitution system. In considering an explanation, we note that the liposome approach uses artificial membranes, while the transport assay and the reconstitution system study COPI transport in the context of Golgi membrane. Thus, as native membranes are more complex than artificial membranes, a likely explanation is that one or more lipids/proteins that exist in the native membrane, but not in the liposomal membrane, prevent the polyunsaturated forms from having a role in COPI vesicle fission.

Studies thus far on vesicle fission across different intracellular pathways have been largely protein-centric^1–4^. As such, our elucidation of how key proteins and lipids coordinate their actions to drive COPI vesicle fission advances a fundamental understanding of the fission process. Because we have found that late fission involves the sequential actions of short PA followed by short DAG, we propose an overall model whereby BARS cooperates with short PA to drive late fission initially, and then ARF1 and ARFGAP1 cooperate with short DAG to drive late fission to completion (summarized in Supplementary Fig. 10). Finally, as basic mechanisms of vesicular transport are conserved, it will be interesting to see in the future whether shortened acyl chains constitute a form of lipid geometry that is universally needed for membrane fission in intracellular pathways.

## Methods

### Chemicals and lipids

The following chemicals and lipids were used: guanine nucleotide triphosphate (GTP) and protease inhibitor cocktail from Sigma-Aldrich; 2C6:0 PA (dihexanoylphosphatidic acid, 830841), 2C10:0 PA (didecanoylphosphatidic acid, 830843), 2C18:0 PA (distearoylphosphatidic acid, 830865), 2C18:1 PA (dioleoylphosphatidic acid, 840875), C18:0-22:6 PA (1-stearoyl-2-docosahexaenoylphosphatididc acid, 840864), 2C10:0 DAG (didecanoyldiacylglycerol, 800810), 2C16:0 DAG (dipalmitoyldiacylglycerol, 800816), 2C18:1 DAG (dioleoyldiacylglycerol, 800811), C18:0-22:6 DAG (1-stearoyl-2-docosahexaenoyldiacylglycerol, 800819), 2C18:1 PC (dioleoylphosphatidylcholine (DOPC), 850374), 2C18:1 PE (dioleoylphosphatidylethanolamine (DOPE), 850725), 2C18:1 SM (dioleoylsphingomyelin, 860587), 2C18:1 PI (dioleoylphosphatidylinositol (DOPI), 850149), and Cholesterol (700000), all from Avanti Polar Lipid; and protein G-agarose bead from Santa Cruz Biotechnology (sc-2002).

### Proteins

Preparations of ARF1, ARFGAP1, BARS, and coatomer have been described in detail^38^. Briefly, myristoylated ARF1 was generated through bacterial expression. BL21 bacteria were transformed with two plasmids, one encoding *ARF1* in pET3 and the other encoding *N-myristoyltransferase* in pBB131. After expression, the cell lysate was subjected to sequential purification using HiTrap Q HP column, a HiPrep 26/60 Sephacryl S-100 column, and then a HiTrap phenylsepharose column. ARFGAP1 was generated using baculovirus expression. Briefly, his-tagged *ARFGAP1* in pVL1392 plasmid was expressed using the BestBac 2.0 Baculovirus Co-transfection Kit. After expression, the cell lysate was subjected to purification using a Ni-NTA column. BARS was generated by expressing a plasmid encoding his-tagged *BARS* in pET-15b using BL21 bacteria. After expression, the cell lysate was subjected to purification using a Ni-NTA column. Coatomer was purified from rat liver. Briefly, liver was homogenized and then subjected to ammonium sulfate precipitation followed by resuspension for sequential purification using a DEAE-Sepharose FF column, a HiTrap Q HP column, and then a Resource Q column.

### Synthesis of PA and DAG with mixed acyl chain length

See Supplementary Methods (which contain Supplementary Figs. 11–24) for details. Synthesis of enantiopure mixed DAGs is challenging due to facile 1,2-acyl shift of the fatty acid residues, thereby releasing steric compression. We previously reported a strategy to suppress this undesired reaction^39^, and this method was used to prepare the desired lipids. In brief, enantiopure silyl-protected glycidol was subjected to a Jacobsen-type ring-opening reaction using palmitic acid as the nucleophile. The resulting secondary hydroxyl group was esterified using Steglich conditions with decanoic acid (capric acid) in an overall 82% yield. In parallel, this sequence was carried out, now with capric acid as the nucleophile and palmitic acid in the esterification reaction. Desilylation was accomplished by short treatment with BF3 acetonitrile complex, followed by quenching with ice-cold aqueous phosphate buffer and subsequent extraction, to afford the desired DAGs. These products were reacted with dibenzyl *N*,*N*-diisopropylphosphoramidite to produce the phosphites, which were in situ oxidized to the corresponding phosphates. Hydrogenolysis provided the desired PAs as their triethylammonium salts after purification by flash chromatography on silica gel.

### Antibodies

Mouse antibodies against β-COP (M3A5, 1:10 dilution western blotting (WB)), VSVG (BW8G65, 1:5 dilution immunofluorescence (IF)), the Myc epitope (9E10, 1:10 dilution WB), and rabbit antibodies against ARF1 (1:1000 dilution WB), BARS (1:1000 dilution WB), cellubrevin (1:1000 dilution WB), giantin (1:1000 dilution IF), LPAAT-γ (1:1000 dilution WB), and PLD2 (1:1000 dilution WB) have been described^14–16,40^. Mouse antibody against LPP3 was obtained (Abcam, AB52581, 1:1000 dilution WB). Antibodies obtained from Cell Signaling Technology were: rabbit anti-HA epitope (3724S, 1:1000 dilution WB), horse anti-mouse IgG conjugated with horseradish peroxidase (HRP; 7076S, 1:5000 dilution WB), and goat anti-rabbit IgG conjugated with HRP (7074S, 1:5000 dilution WB). Other antibodies were obtained from Jackson ImmunoResearch Laboratory: donkey anti-rabbit IgG conjugated with Alexa Fluor 488 (711-545-152, 1:250 dilution IF) and Donkey anti-mouse IgG conjugated with Alexa Fluor 594 (715-585-150, 1:250 dilution IF). Uncropped blots for Supplementary figures are provided in the Source data file.

### Plasmids and siRNA

Plasmids encoding for *PLD2*, HA-tagged *LPP3*, and Myc-tagged *BARS* have been described^15,16,18^. The following sequences were used for siRNA experiments: siRNA against *PLD2* (5′-GGACAACCAAGAAGAAAUA-3′), siRNA against *LPAAT-γ* (5′-GAGACCAAGCACCGCGUUA-3′), and siRNA against *LPP3* (5′-GGGACUGUCUCGCGUAUCA-3′). Efficacy of these siRNA sequences has been previously established^15,16,18^. To assess the efficiency of siRNA  transfection, a Cy3-conjugated scrambled sequence was used (5′-GACGCUGGUUCACCAUUCA-3′).

### Cells

HeLa cells, obtained from the ATCC (American Type Culture Collection) and certified as free of mycoplasma contamination, were cultured in Dulbecco’s modified essential medium supplemented with 10% fetal bovine serum, 2 mM glutamine, and 40 µg ml^−1^ gentamicin. Transfection of DNA plasmids was performed using FuGene6 (Promega). Transfection of siRNA was performed using Lipofectamine^®^ RNAiMAX (Invitrogen).

### COPI vesicle reconstitution system

The two-stage incubation system has been described in detail^38^. Briefly, Golgi membrane (100 µg) was pre-washed with 3 M KCl for 5 min and then resuspended with reaction buffer (25 mM HEPES-KOH, pH 7.2, 50 mM KCl, 2.5 mM magnesium acetate, 1 mg ml^−1^ soybean trypsin inhibitor, 1 mg ml^−1^ bovine serum albumin (BSA), and 200 mM sucrose). For the first stage, the washed membrane was incubated with ARF1, coatomer, and GTP at 37 °C for 15 min. Reaction was stopped on ice for 5 min, followed by centrifugation at 12,000 × *g* for 10 min. For the second stage, the pellet was resuspended again in reaction buffer and then incubated with ARFGAP1 and BARS for 20 min.

To study the different stages of COPI vesicle fission, as defined by the different lipid enzymes (summarized in Supplementary Fig. 10), Golgi membrane was isolated from cells that had been treated with siRNA against different lipid enzymes to inhibit different stages of fission, followed by functional rescue using the lipid product of that enzyme. To examine early fission, Golgi membrane was collected from cells treated with siRNA against *LPAAT-γ*, and then functional rescue was performed by adding liposomes that contain PA of various forms (200 µM, with DOPC:PA in 1:1 molar ratio) to the reconstitution system (after the first-stage incubation) for 10 min at 37 °C. To examine late fission mediated by PLD2, Golgi membrane was collected from cells treated with siRNA against *PLD2*, and then functional rescue was performed by adding liposomes that contain PA of various forms (200 µM, with DOPC:PA in 1:1 molar ratio) to the reconstitution system (after the first-stage incubation) for 10 min at 37 °C. To examine late fission mediated by LPP3, Golgi membrane was collected from cells treated with siRNA against *LPP3*, and then functional rescue was performed by adding liposomes that contain PA of various forms (200 µM, with DOPC:DAG in 1:1 molar ratio) to the reconstitution system (after the first-stage incubation) for 10 min at 37 °C. The number of experiments performed is indicated in the figure legend.

### COPI transport assay

Quantitation of retrograde transport of the COPI-dependent cargo, VSVG-ts045-KDELR, from the Golgi to the ER has been described^19,40^. Briefly, Hela cells were transfected with VSVG-ts045-KDELR. Cells were then incubated at 40 °C for 2 h, which accumulates a synchronized pool of the cargo at the ER, and then incubated at 32 °C for 2 h for the synchronized accumulation of the cargo at the Golgi. Cells were then incubated at 40 °C for one round of retrograde transport of the cargo from the Golgi to the ER. Cells were then examined randomly to assess the colocalization of VSVG-ts045-KDELR with a Golgi marker (giantin). Images were acquired using either the Nikon EZ-C1 version 3.90 acquisition software or the Zeiss Zen 2.3 blue edition confocal acquisition software. Colocalization was quantified using the NIH Image J software or Metamorph software.

For the delivery of a specific lipid into cells, the lipid was mixed with DOPC (1:1 molar ratio), dried, and then rehydrated in culture medium that contained BSA (25 µM) to achieve a final lipid concentration of 125 µM in the culture medium. For each condition of an experiment, five cells were examined. The number of experiments performed is indicated in the figure legend.

### Liposome deformation assay

To generate liposomes, lipids (200 µg) in glass tubes are evaporated under N_2_ gas. Dried lipids were then resuspended with 200 µl of assay buffer (25 mM HEPES-KOH, pH 7.2, 50 mM KCl, and 2.5 mM magnesium acetate) and hydrated overnight. After freeze/thaw for three times, liposomes were passed through 400-nm filter membrane (Whatman) in a mini-extruder (Avanti Polar Lipids).

Liposomes that mimic the lipid composition of Golgi membrane contain 50% DOPC, 10% DOPE, 7% DOPS, 6% DOPI, 17% cholesterol, 8% SM, 1% PA, and 1% DAG. Simplified liposomes contained 30% PA or DAG along with 70% DOPC. COPI fission factors as recombinant proteins (1 µM) were incubated with liposomes (20 µg) in 100 µl of assay buffer (25 mM HEPES-KOH, pH 7.2, 50 mM KCl, and 2.5 mM magnesium acetate) at 25 °C for 30 min. Samples were then fixed using 2% paraformaldehyde (PFA)/phosphate-buffered saline (PBS) for 10 min, followed by EM analysis. To score for vesiculation by COPI fission factors, we quantify the number of rounded membrane structures in the range of 30–50 nm in diameter. In comparison, liposomes are typically in the range of 100–400 nm in diameter, and membrane structures in this range are scored as no vesiculation. Tubulation is scored as liposomes that contain protrusive rod-like structures.

In the case of ARF1, GTP was first loaded onto ARF1 (20 µg) at 32 °C for 90 min in 100 µl of nucleotide loading buffer (2.5 mM HEPES-KOH, pH 7.2, 100 mM NaCl, 1 mM DTT, 1 mM EDTA, 1 mM MgCl_2_, and 2 mM GTP). The reaction was then stopped by adjusting MgCl_2_ to 5 mM.

### Whole-mount EM analysis

EM examination of COPI buds on Golgi membrane has been described^14^. In brief, membrane samples from the COPI reconstitution system were spotted onto EM grids and then fixed with 2% PFA/PBS for 10 min. Grids were rinsed three times with water, followed by 1% uranyl acetate staining, and then examined using JEOL 1200EX transmission electron microscope.

To assess the degree of liposome tubulation and vesiculation induced by the COPI fission factors, samples were spotted onto EM grids. Grids were rinsed three times with water, followed by 1% uranyl acetate staining. Fifty EM meshes were then examined for quantitation in each condition of an experiment. Vesiculation was quantified by counting vesicles with diameter <50 nm. Tubulation was quantified by counting liposomes with linear protrusions. The number of experiments performed is indicated in the figure legend.

### Liposome cryo-vitrification and cryo-EM

A drop (3 µl) of the liposome solution was applied onto a 300-mesh QUANTIFOIL R2/1 100 holy carbon grid that was pretreated for 1 min in a plasma cleaner using Gatan Solarus 950 with a mixture gas of H_2_ and O_2_. Cryo-vitrification was performed using ThermoFisher Scientific Vitrobot IV. The grid was then blotted for 4.0 s with a blot force 0 at 100% humidity and 22 °C before it was quickly frozen in liquid ethane that was cooled by liquid nitrogen. Cryo-vitrified grids were imaged using a ThermoFisher Scientific Talos F200C cryo-electron microscope that was operated under 200 kV. Low-dose images (15 e Å^−2^) with a magnification of 36,000 were collected using SerialEM. The defocus was set at 1 µm.

### CG MD simulations

All simulations were performed using GROMACS 5.1.4 (www.gromacs.org) package^41^ and the MARTINI CG force field^42,43^. Each MD model contains a bilayer of 16,900 lipids, with 70 mol% DOPC and 30 mol% of different PA/DAG forms, PA (2C10:0), PA (2C18:0), DAG (2C10:0), or DAG (2C18:0). The lipid bilayer was solvated in ~3,500,000 water particles to ensure considerable box size in the direction perpendicular to the membrane surface. Sodium counterions were added to PA systems to neutralize the models. Before applying a constant force (350 kJ mol^−1^ nm^−1^) to a lipid membrane patch (with radius of ~30 Å) along the perpendicular direction, each membrane was first relaxed for 200 ns to reach the equilibrium state and then continuously simulated for another 200 ns to calculate membrane properties and evaluate errors. Under the pulling force, membranes undergo tubulation and then stop when the size limitation of the models was reached. Simulation of membrane deformation for each system was replicated four times, with each time applying constant force to a different portion of the membrane, which generated similar results.

As employed in previous membrane deformation/tethering simulations^23^, the standard cutoffs for MARTINI force field were implemented. The lateral and normal pressures were coupled to 1 bar with semi-isotropic pressure coupling scheme under the Berendsen barostat^44^. The temperature of lipids and aqueous solutions was separately coupled to 310 K using the velocity rescaling thermostat^45^. Model images were generated and rendered using the Visual Molecular Dynamics software^46^.

### Calculations of membrane properties and force

To calculate membrane thickness, each membrane model was divided into 40 × 40 grids in the *x*–*y* plane. The center-of-mass of phosphorus atoms in the upper and bottom leaflets in each grid were used to calculate and average the thickness. The lateral diffusion coefficient (*D*_L_) of lipids was plotted from mean square displacement of the center-of-mass of lipids using a locally written code. The bending rigidity (*K*_C_) of lipid membranes was calculated using the code developed by Fowler et al.^47^, which is based on Helfrich–Canham theory that relates the elastic properties of lipid membranes to their surface fluctuation.

Potential of mean force (PMF profiles) were calculated using umbrella sampling^48,49^ and Weighted Histogram Analysis Method^50^. Umbrella sampling windows were partitioned along the direction normal to membrane planes with an interval of 1 Å, going from the bilayer center to the bilayer surface. The headgroup of a PA/DAG lipid was restrained to each window with a harmonic force constant of 2000 kJ mol^−1^ nm^−2^. Each window was sampled for 100 ns, and distance distribution histograms were collected within time range 50–70, 60–80, 70–90, and 80–100 for PMF calculation and error evaluation.

### MS of Golgi lipids

To detect endogenous PC on Golgi membrane, 5 mg of this membrane fraction was extracted using acidified CHCl_3_/methanol (1:2) and analyzed using an Ultimate 3000 ultra-high-performance liquid chromatography system coupled to a Thermo Q-Exactive Orbitrap mass spectrometer (Thermo Scientific). Lipid extracts were separated on a Waters ACQUITY BEH C8 column (2.1 × 100 mm, 1.7 μm) with the temperature maintained at 40 °C. The flow rate was 250 μl min^−1^, and the mobile phases consisted of 60:40 water/acetonitrile (A) and 90:10 isopropanol/acetonitrile (B), both containing 10 mM ammonium formate and 0.1% formic acid. The samples were eluted with a linear gradient from 5% B to 97% B over 20 min, maintained at 97% B for 4 min and re-equilibration with 5% B for 6 min. The sample injection volume was 5 μl. The mass spectrometer was operated in positive ionization mode. The full scan and fragment spectra were collected at a resolution of 70,000 and 35,000, respectively. Data analysis and accurate mass calculation were performed using the software Xcalibur 4.0 (Thermo Fisher).

To detect PA and DAG on Golgi membrane from cells fed with short PA, we performed quadrupole time of flight (QTOF) MS detection of lipids. Golgi-derived samples prepared as above were diluted (1:2) in high-performance liquid chromatography (HPLC)-grade methanol and resolved using two related HPLC-MS/MS methods using reversed phase elution on an Agilent Poroshell 120, C18, 5 cm × 3 mm column and detected with an Agilent 6420 QTOF LCMS system with standard duel electrospray source and 20 V collision energy. Didecanoyl glycerol was analyzed with positive mode MS/MS ion monitoring (*m*/*z* 418 to *m*/*z* 229.180) using a binary gradient with solvent A (methanol, 5% water, 2 mM ammonium formate) and solvent B (1-propanol, 10% cyclohexane, 2 mM ammonium formate) combined in multiple stages (0–4 min 0% B, 4–13 min 0–100% B on a linear gradient, 13–18 min 100% B, 18–20 min 100% B to 0% B on a linear gradient, 20–30 min 0% B). Didecanoyl PA was analyzed in negative mode by monitoring the transition from *m*/*z* 479 to *m*/*z* 171.143 using a gradient that is identical to the above except solvent A is 10% water in methanol with 2 mM ammonium formate. This change was made to achieve higher separation of this phospholipid from other early-eluting compounds.

### Statistical analysis

Sample size is noted in the figure legends. Sample size used was based on our previous familiarity with the assays. Statistical significance was determined using the Excel or Prism software for the two-tailed Student’s *t* test. No inclusion/exclusion criteria were pre-established. The experiments were not randomized. The investigators were not blinded to the group allocation during experiments and in outcome assessment.

### Reporting summary

Further information on research design is available in the Nature Research Reporting Summary linked to this article.

## Supplementary information

Supplementary information_new

Reporting Summary

Source Data

## Data Availability

The authors declare that all data supporting the findings of this study are available within the article and its supplementary information files or from the corresponding author upon reasonable request. The lipidomics dataset can be accessed at: www.metabolomicsworkbench.org^51^, using project ID PR000789 and study ID ST001177, using the following link: [10.21228/M8PT1R]. The source data underlying Figs. 1a–h, 2a–f, 3a, c–h, 4c–d, 6a–f, 7a–h, 8a–h, 9a–h, Table 1, and Supplementary Figs. 1e–g, 4a, 5a–c, e, g, 6b, d, and 8a–b are provided as a Source Data file.

## Code availability

A locally written code was used to calculate the lateral diffusion coefficient (*D*_L_) of lipids, which can be accessed at a public repository using the following link: (https://github.com/truth-zhenli/MSD_calculation).
