# Supplementary material for: The late stage of COPI vesicle fission requires shorter forms of phosphatidic acid and diacylglycerol
Source: Nat Commun. 2019 Jul 30;10:3409. doi: 10.1038/s41467-019-11324-4 (PMC6667475; doi:10.1038/s41467-019-11324-4)
Supplement: Supplementary file 1 — Supplementary information_new [file 41467_2019_11324_MOESM1_ESM.pdf]

SUPPLEMENTARY INFORMATION

The late stage of COPI vesicle fission requires shorter forms of phosphatidic acid and diacylglycerol

Park et al

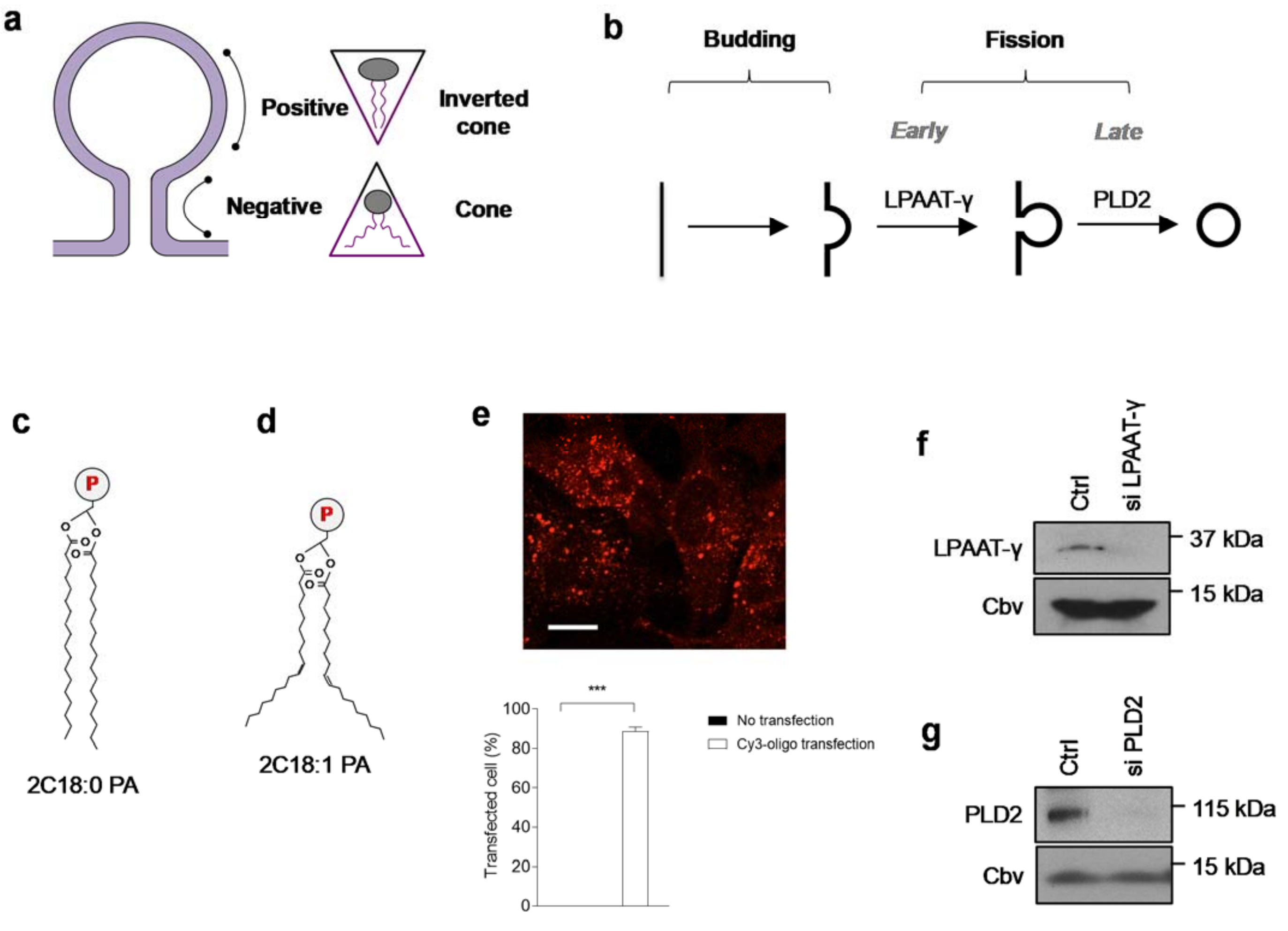

Supplementary Figure 1. Effects of PA in COPI transport. **(a)** Schematic showing the two general types of membrane curvature during vesicle formation, with positive curvature promoted by "inverted-cone" lipid geometry and negative curvature promoted by "cone" lipid geometry. **(b)** Schematic showing the budding and fission stage of COPI vesicle formation, with fission further sub-divided into early versus late, as defined by the roles of different lipid enzymes. **(c)** Schematic showing PA with saturated acyl chains. **(d)** Schematic showing PA with single unsaturation in both acyl chains, having cis double bond at carbon position 9. **(e)** Efficiency of siRNA treatment assessed by the expression of Cy3-labeled scrambled oligonucleotide. Representative image is shown above, bar = 10 uM. Quantitation is shown below, n= 3 independent experiments mean ± s.e.m, two-tailed Student *t*-test, ***P<0.001. Source data are provided as a Source Data file. **(f)** Confirmation of reduced LPAAT−γ level upon siRNA against *LPAAT−γ*. HeLa cells were subjected to the siRNA treatment followed by immunoblotting for LPAAT−γ. **(g)** Confirmation of reduced PLD2 level upon siRNA against PLD2. HeLa cells were subjected to the siRNA treatment followed by immunoblotting for PLD2.

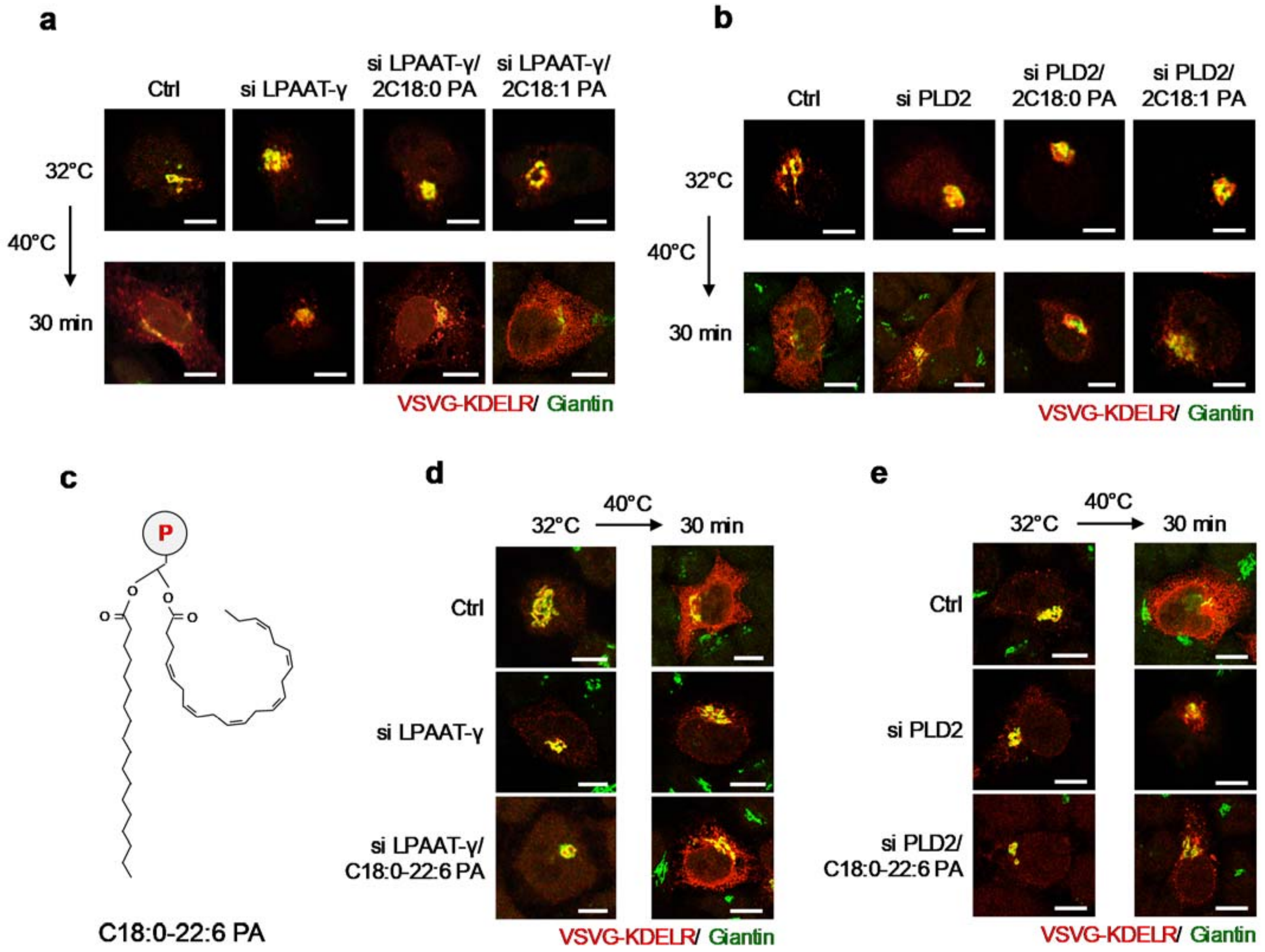

Supplementary Figure 2. Effects of unsaturated PA in COPI transport. **(a)** HeLa cells were treated with siRNA against *LPAAT*$-\gamma$ to inhibit COPI transport, with rescue using different forms of PA as indicated. COPI transport in cells was tracked by examining the colocalization of a COPI-dependent cargo protein (VSVG-KDELR) with Golgi marker (giantin) at time points as indicated. Representative images are shown, bar = 5um. **(b)** HeLa cells were treated with siRNA against *PLD2* to inhibit COPI transport, with rescue using different forms of PA as indicated. COPI transport in cells was tracked by examining the colocalization of a COPI-dependent cargo protein (VSVG-KDELR) with Golgi marker (giantin) at time points as indicated. Representative images are shown, bar = 5um. **(c)** Schematic showing PA with polyunsaturation in one acyl chain, with conformation as suggested by the recent study on clathrin vesicle fission. **(d)** HeLa cells were treated with siRNA against *LPAAT*$-\gamma$ to inhibit COPI transport, with rescue using PA form as indicated. COPI transport in cells was tracked by examining the colocalization of a COPI-dependent cargo protein (VSVG-KDELR) with Golgi marker (giantin) at time points as indicated. Representative images are shown, bar = 5um. **(e)** HeLa cells were treated with siRNA against *PLD2* to inhibit COPI transport, with rescue using PA form as indicated. COPI transport in cells was tracked by examining the colocalization of a COPI-dependent cargo protein (VSVG-KDELR) with Golgi marker (giantin) at time points as indicated. Representative images are shown, bar = 5um.

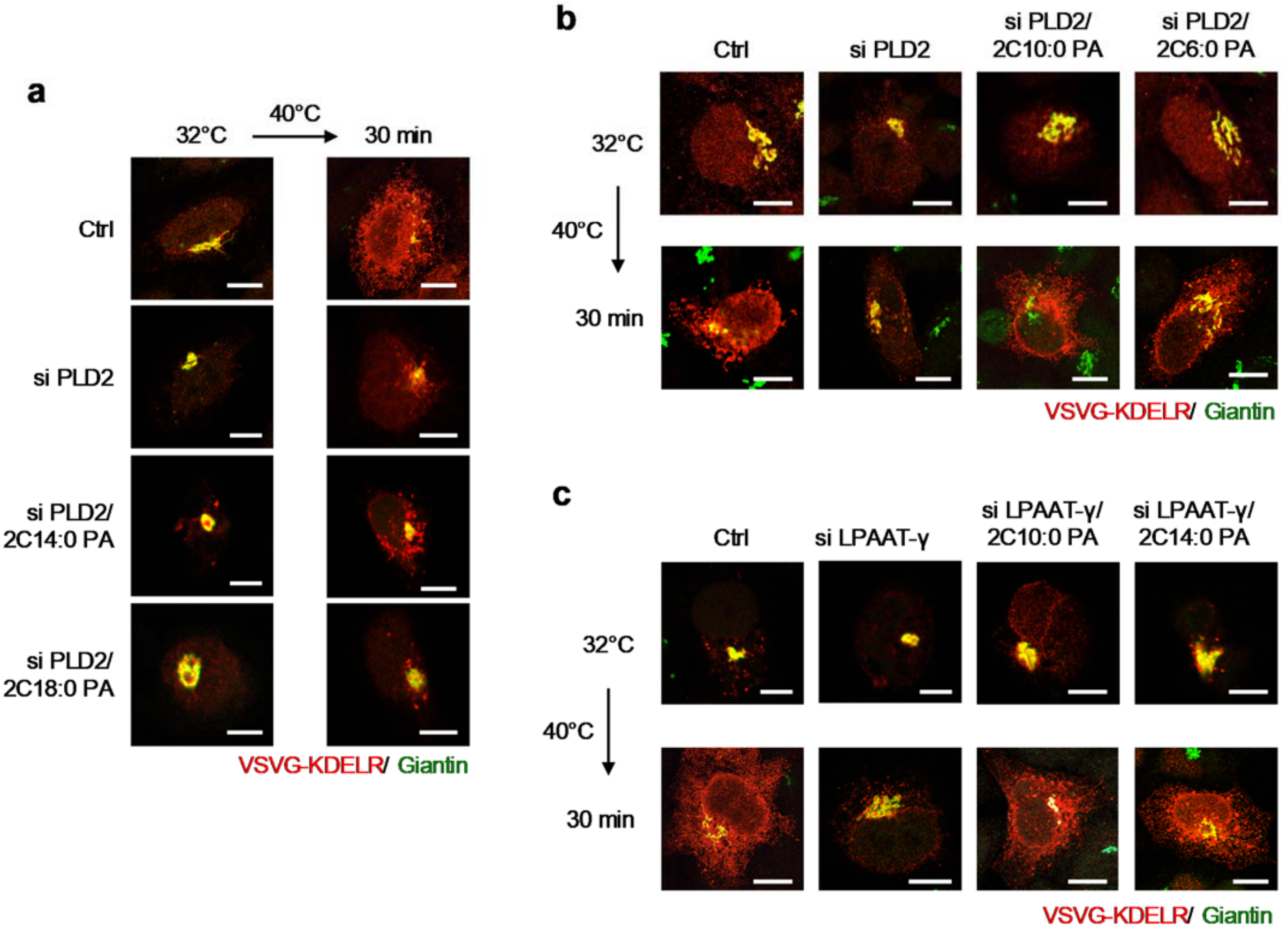

Supplementary Figure 3. Effects of short PA in COPI transport. **(a, b)** HeLa cells were treated with siRNA against *PLD2* to inhibit COPI transport, with rescue using different forms of PA as indicated. COPI transport in cells was tracked by examining the colocalization of a COPI-dependent cargo protein (VSVG-KDELR) with Golgi marker (giantin) at time points as indicated. Representative images are shown, bar = 5um. **(c)** HeLa cells were treated with siRNA against *LPAAT−γ* to inhibit COPI transport, with rescue using different forms of PA as indicated. COPI transport in cells was tracked by examining the colocalization of a COPI-dependent cargo protein (VSVG-KDELR) with Golgi marker (giantin) at time points as indicated. Representative images are shown, bar = 5um.

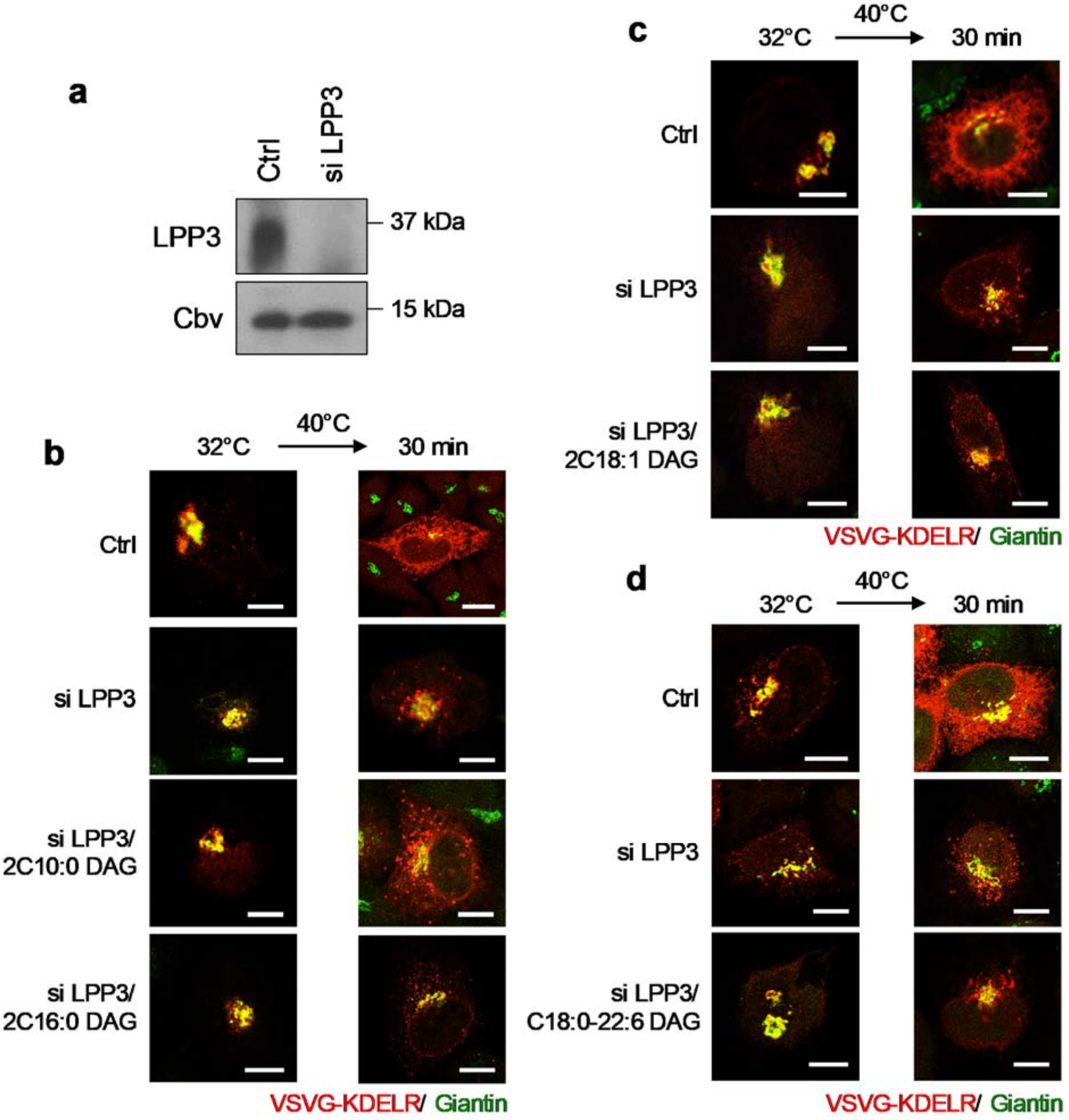

Supplementary Figure 4. Effects of DAG in COPI transport. **(a)** Confirmation of reduced LPP3 level upon siRNA against *LPP3*. HeLa cells were subjected to the siRNA treatment followed by immunoblotting for LPP3. Note that LPP3 appears as multiple bands due to its glycosylation. **(b)** HeLa cells were treated with siRNA against *LPP3* to inhibit COPI transport, with rescue using different forms of DAG as indicated. COPI transport in cells was tracked by examining the colocalization of a COPI-dependent cargo protein (VSVG-KDELR) with Golgi marker (giantin) at time points as indicated. Representative images are shown, bar = 5um. **(c)** HeLa cells were treated with siRNA against *LPP3* to inhibit COPI transport, with rescue using DAG form as indicated. COPI transport in cells was tracked by examining the colocalization of a COPI-dependent cargo protein (VSVG-KDELR) with Golgi marker (giantin) at time points as indicated. Representative images are shown, bar = 5um. **(d)** HeLa cells were treated with siRNA against *LPP3* to inhibit COPI transport, with rescue using DAG form as indicated. COPI transport in cells was tracked by examining the colocalization of a COPI-dependent cargo protein (VSVG-KDELR) with Golgi marker (giantin) at time points as indicated. Representative images are shown, bar = 5um.

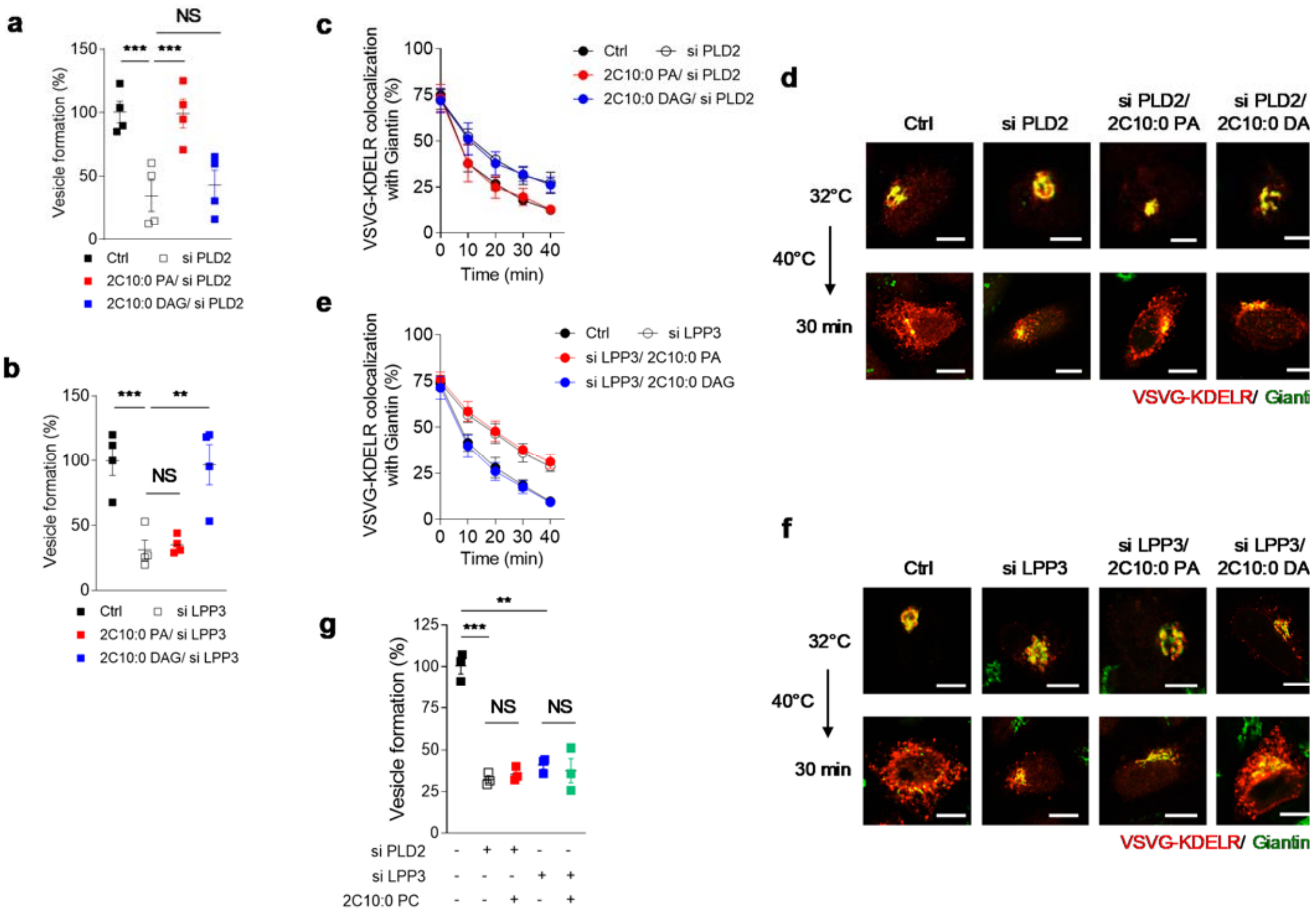

Supplementary Figure 5. PA and DAG have distinct roles in COPI transport. Quantitative data are shown as mean ± s.e.m. Significance was tested using the two-tailed Student *t*-test, ** P<0.01, *** P<0.001, NS P>0.05. Source data are provided as a Source Data file.

(a) Golgi membrane with reduced PLD2 level was used for the vesicle reconstitution system, with rescue using either short PA or DAG; n=5 independent experiments. **(b)** Golgi membrane with reduced LPP3 level was used for the vesicle reconstitution system, with rescue using either short PA or DAG; n=5 independent experiments. **(c)** HeLa cells were treated with siRNA against *PLD2* with rescue using either short PA or DAG. COPI transport was then quantified, n=4 independent experiments. **(d)** HeLa cells were treated with siRNA against *PLD2* with rescue using either short PA or DAG. Colocalization of a COPI-dependent cargo protein (VSVG-KDELR) with Golgi marker (giantin) was then assessed. Representative images are shown, bar = 5um. **(e)** HeLa cells were treated with siRNA against *LPP3* with rescue using either short PA or DAG. COPI transport was then quantified, n=4 independent experiments. **(f)** HeLa cells were treated with siRNA against *LPP3* with rescue using either short PA or DAG. Colocalization of a COPI-dependent cargo protein (VSVG-KDELR) with Golgi marker (giantin) was then assessed. Representative images are shown, bar = 5um. **(g)** Golgi membrane with reduced PLD2 or LPP3 level was used for the vesicle reconstitution system. Rescue was performed using short PC; n=3 independent experiments.

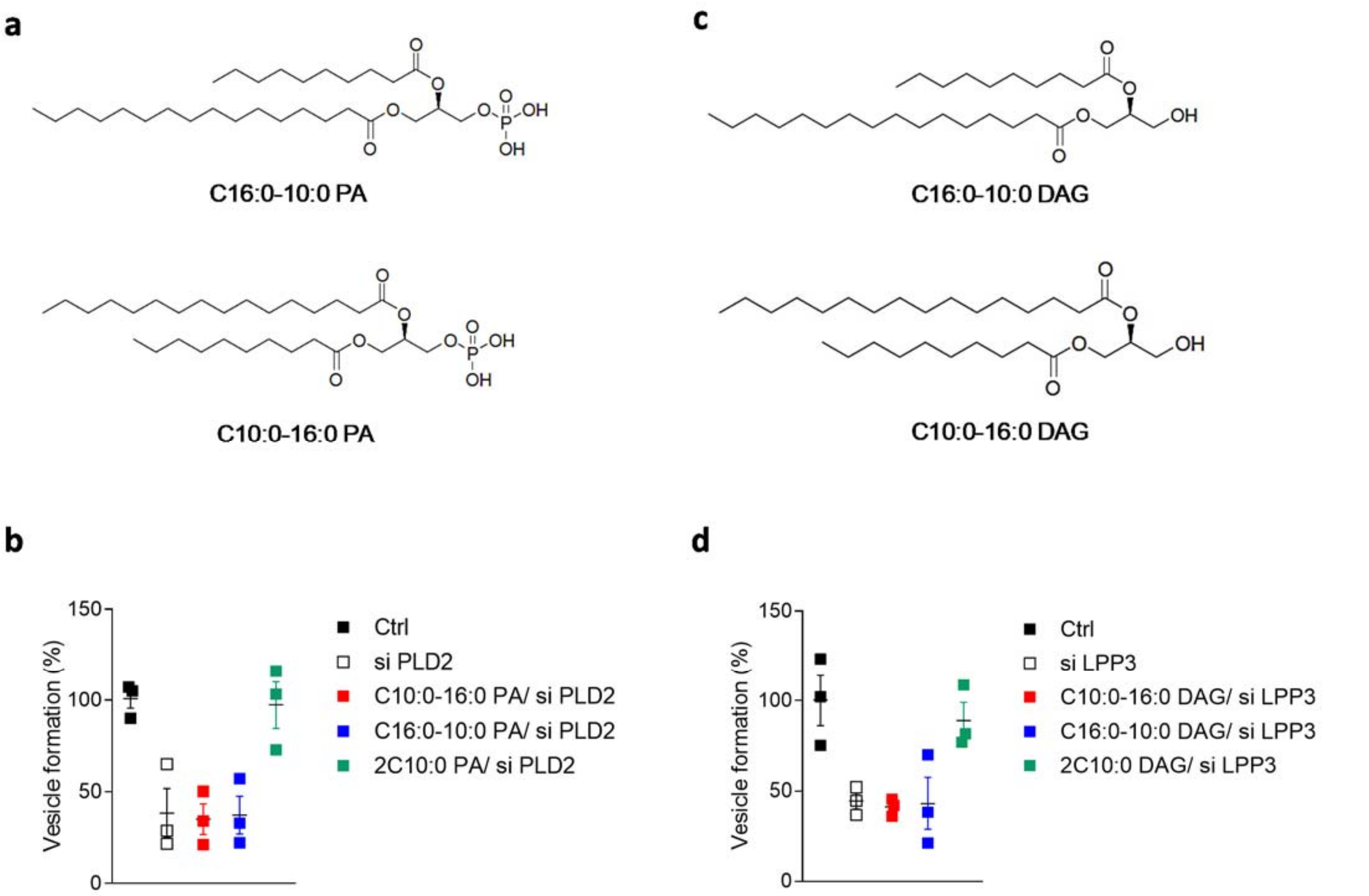

Supplementary Figure 6. PA and DAG with mixed acyl-chain lengths in COPI vesicle formation. Quantitative data are shown as mean ± s.e.m. Source data are provided as a Source Data file.
(a) Schematic of PA with mixed acyl-chain lengths. **(b)** Golgi membrane with reduced PLD2 level was used for the COPI vesicle reconstitution system, with rescue of vesicle formation using forms of PA as indicated; n=3 independent experiments. **(c)** Schematic of DAG with mixed acyl-chain lengths. **(d)** Golgi membrane with reduced LPP3 level was used for the COPI vesicle reconstitution system, with rescue of vesicle formation using forms of DAG as indicated; n=3 independent experiments.

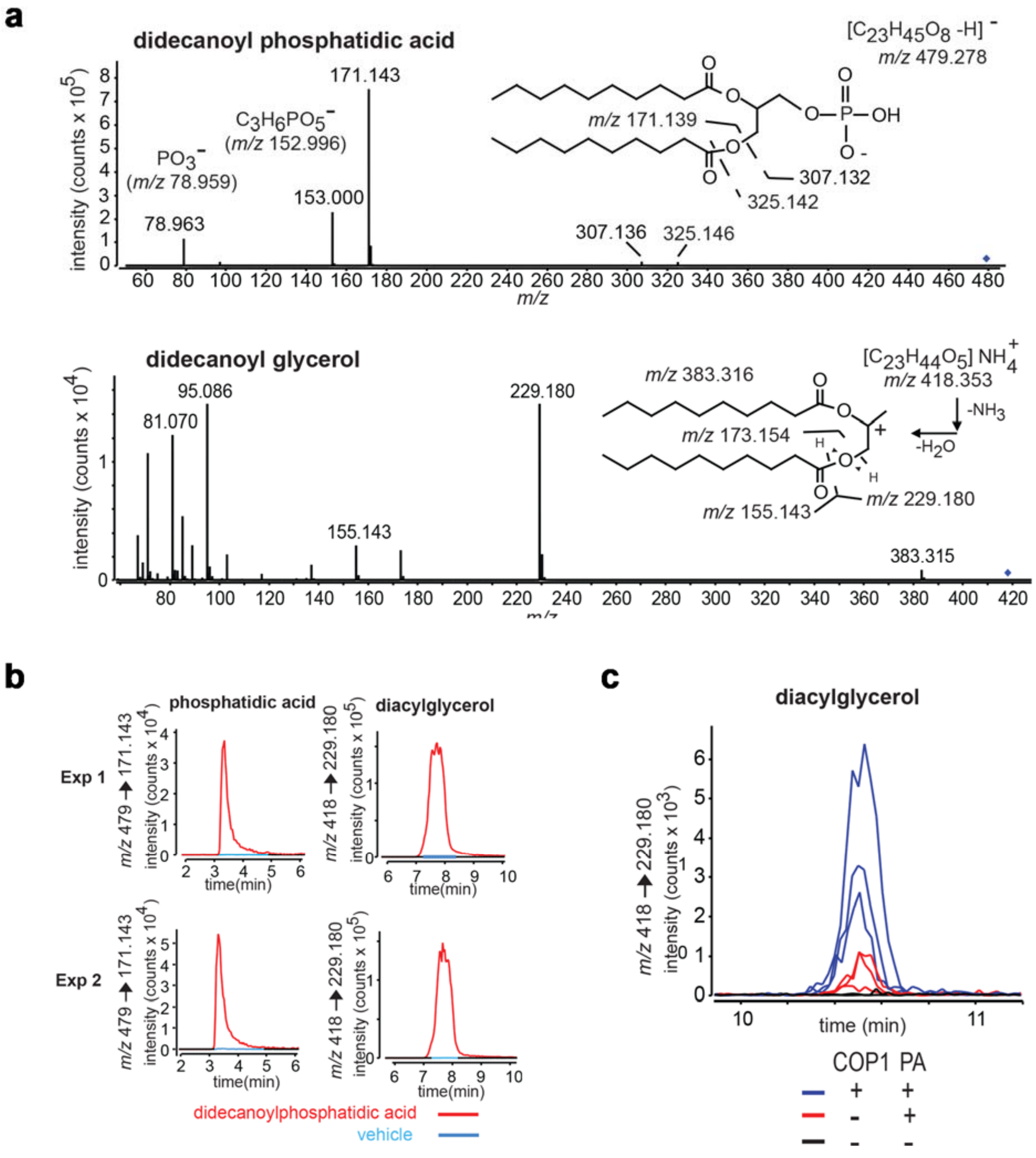

Supplementary Figure 7. Analysis of Golgi membrane from cells fed with short PA. HeLa cells were fed with short (2C10:0) PA, and then Golgi membrane was isolated. After lipid extraction, quadrupole time of flight MS/MS detection was performed. **(a)** Collision induced dissociation mass spectrometry of PA in the negative mode and DAG in the positive mode show the expected fragmentation patterns, including diagnostic ions for decanoyl PA (m/z 479 to 171) and decanoyl DAG (m/z 418 to 229), which were used to track these compounds in subsequent cellular experiments. **(b)** Complex cellular lipids eluting into methanol were injected followed by specific tracking of PA and DAG based on the indicated MS/MS signals. Two representative experiments (out of five) are shown. **(c)** Golgi membrane subjected to the COPI vesicle reconstitution system shows enhanced conversion of short PA to short DAG. A representative experiment (out of two) with three replicates is shown, with each set comparing PA-fed Golgi membrane either without or with incubation in the COPI reconstitution system.

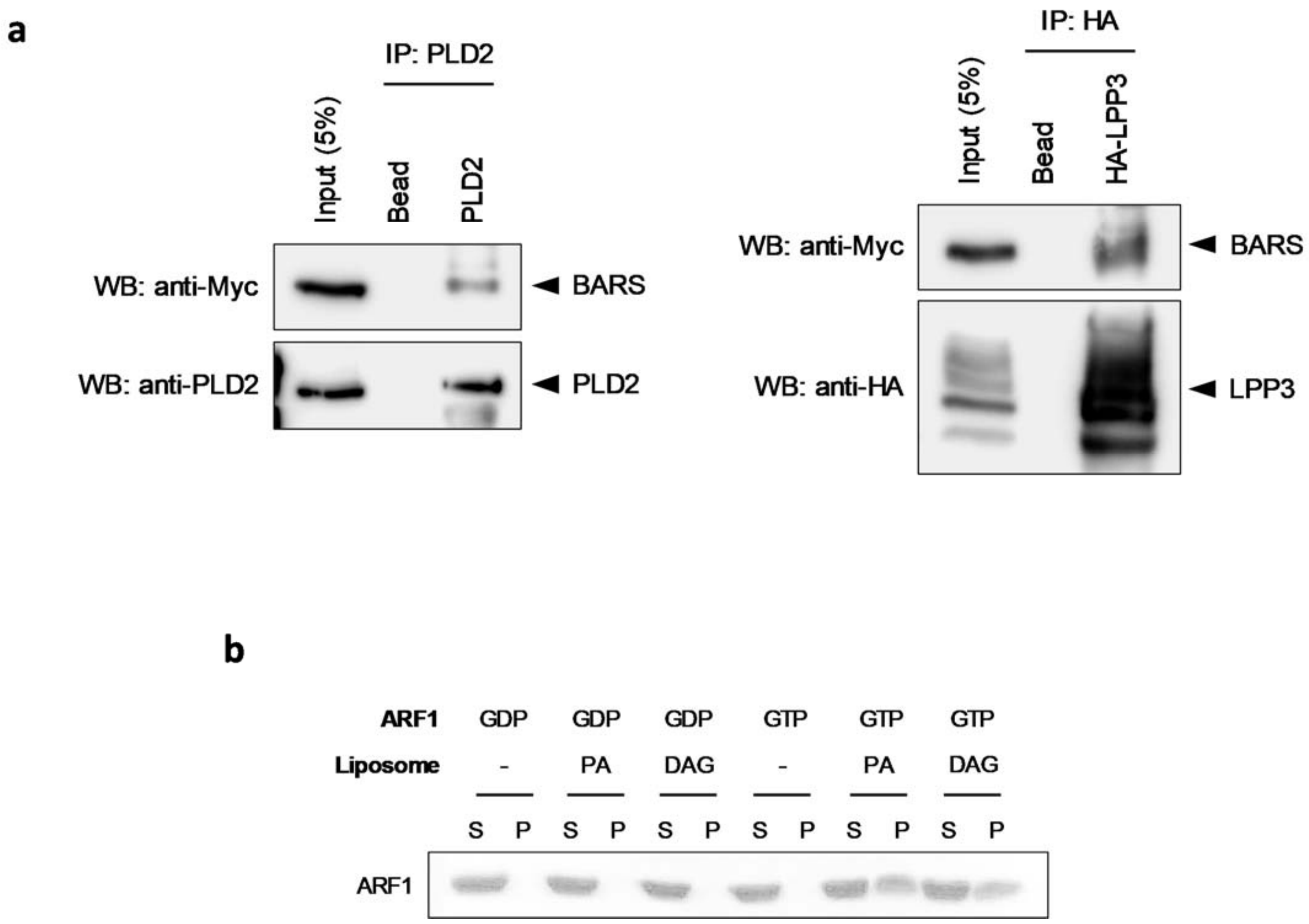

Supplementary Figure 8. Lipid enzymes associate with COPI-related factors. (a) BARS associate with PLD2 and LPP3. Whole cell lysates from HeLa cells were immunoprecipitated for either PLD2 or LPP3 followed by immunoblotting for BARS. Immunoblotting for PLD2 or LPP3 confirms the presence of these enzymes in the immunoprecipitated. HA-tagged form of LPP3 was used because antibody against endogenous LPP3 was inefficient for immunoprecipitation. (**b**) The GTP-bound form is required for ARF1 to bind simplified liposomes. ARF1 in either GTP or GDP form was incubated with different simplified liposomes as indicated, followed by centrifugation to obtain pellet (P, membrane-bound) or soluble (S) fractions.

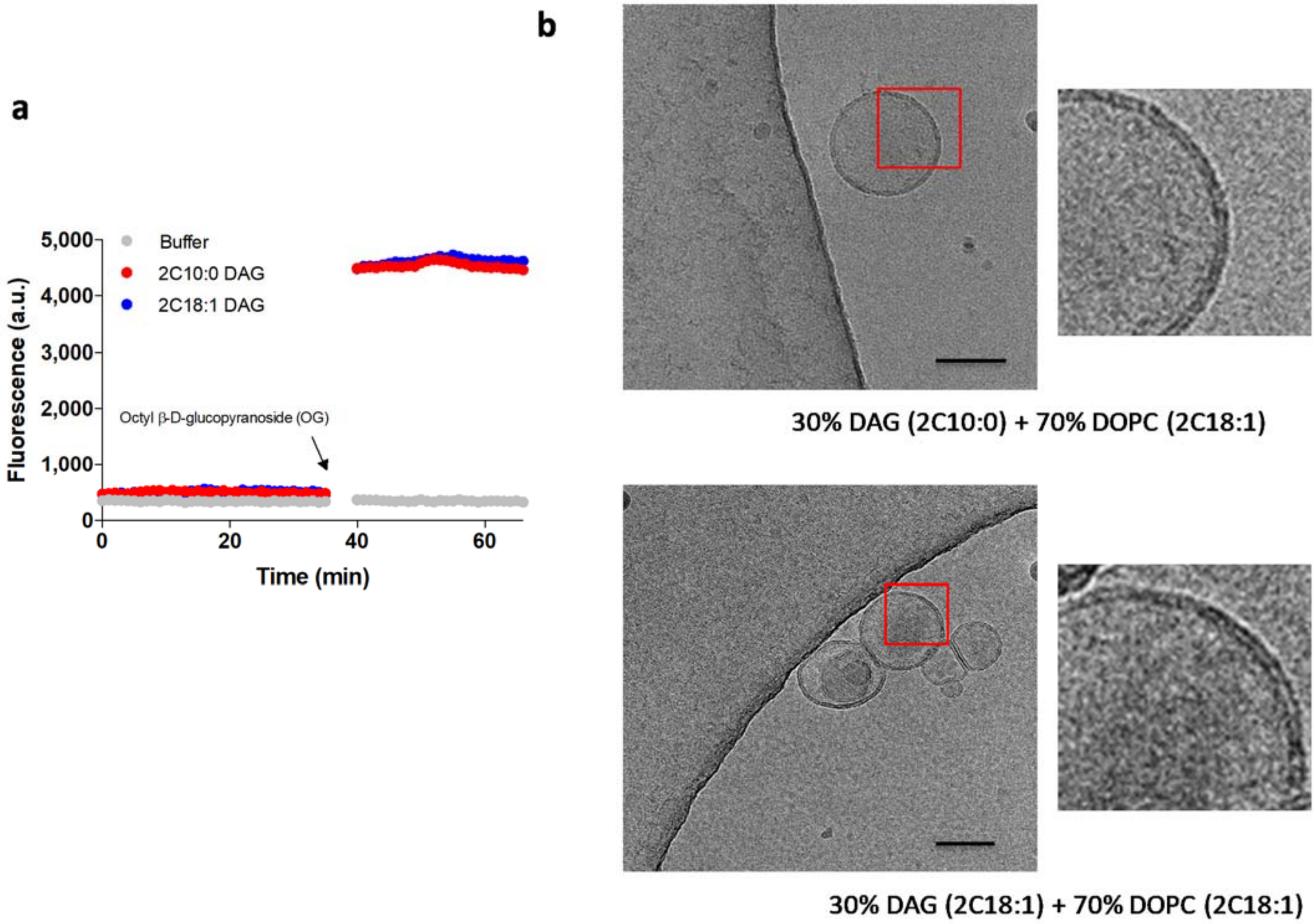

Supplementary Figure 9. Characterization of liposomes. (a) Liposome leakage assay confirming that simplified liposomes are functionally intact. Liposomes with 70% DOPC and 30% of DAG form as indicated were loaded with calcium, and then monitored for leakage out of liposomes using fluo-4, a calcium-sensitive fluorophore, which was placed outside of liposomes. No significant leakage was observed until a detergent (Octyl β-D-glucopyranoside) was added to the incubation. (**b**) High-resolution cryo-EM confirming that simplified liposomes, with lipid composition indicated, form membrane bilayers, bar = 100 nm. A higher magnification of the inset area is shown on right.

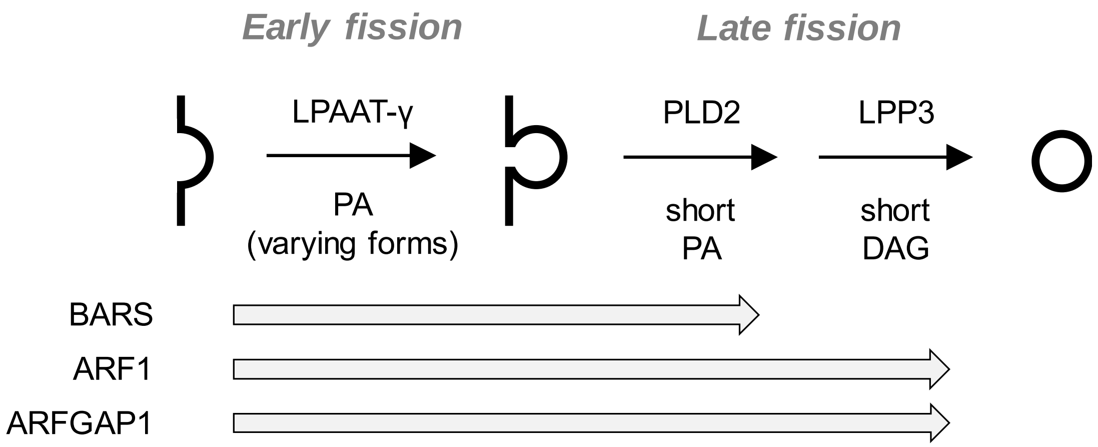

Supplementary Figure 10. Key proteins and lipids driving COPI vesicle fission. Our results suggest that PA of varying forms generated by LPAAT-γ promotes early fission, while short PA generated by PLD2 followed by short DAG generated by LPP3 promote late fission. Moreover, BARS acts in conjunction with PA to deform membrane, with short PA best promoting the vesiculation of BARS, while ARF1 and ARFGAP1 act in conjunction with PA and DAG to deform membrane, with short DAG best promoting the vesiculation abilities of ARF1 and ARFGAP1.

Supplementary Methods

All reactions were carried out under a nitrogen atmosphere using oven-dried glassware and using standard Schlenk techniques. Reaction temperature refers to the temperature of the oil bath. All reagents and catalysts were purchased from Sigma-Aldrich, Acros, J&K Scientific and TCI Europe and used without further purification unless otherwise mentioned, any purification of reagents was performed following previously reported methods[1]. TLC analysis was performed on Merck silica gel 60/Kieselguhr F254, 0.25 mm. Compounds were visualized using either Seebach's reagent (a mixture of phosphomolybdic acid (25 g), cerium (IV) sulfate (7.5 g), H_2O (500 mL) and H_2SO_4 (25 mL)), 2,4-DNP stain (2,4-dinitrophenylhydrazine (12 g), conc. sulfuric acid (60 ml), water (80 ml), ethanol (200 ml)), bromocresol green (a mixture of bromocresol green (0.04 g) in EtOH (100 mL), 0.1 M NaOH added until mixture turns blue), Phosphomolybdic acid (PMA) stain (a mixture of phosphomolybdic acid (10 g) in EtOH (100 mL)) or elemental iodine. Flash chromatography was performed using SiliCycle silica gel type SiliaFlash P60 (230 – 400 mesh).GC-MS measurements were performed with an HP 6890 series gas chromatography system equipped with an HP1 or HP5 column (Agilent Technologies, Palo Alto, CA), and equipped with an HP 5973 mass sensitive detector. High resolution mass spectra (HRMS) were recorded on a Thermo Scientific LTQ Orbitrap XL. (ESI+, ESI- and APCI). ^{1}H-, ^{13}C- and ^{31}P-NMR spectra were recorded on a Varian AMX400 (400, 100.6 and 162 MHz, respectively) using $CDCl_3$ as solvent unless stated otherwise. Chemical shift values are reported in ppm with the solvent resonance as the internal standard ($CDCl_3$: δ 7.26 for ^{1}H, δ 77.16 for ^{13}C). Data are reported as follows: chemical shifts (δ), multiplicity (s = singlet, d = doublet, dd = double doublet, ddd = double double doublet, td = triple doublet, t = triplet, q = quartet, b = broad, m = multiplet), coupling constants J (Hz), and integration. Enantiomeric excesses were determined by chiral HPLC analysis using a Shimadzu LC- 10ADVP HPLC instrument equipped with a Shimadzu SPD-M10AVP diode-array detector. Optical rotations were measured on a Schmidt+Haensch polarimeter (Polartronic MH8) with a 10 cm cell (c given in g/mL) at ambient temperature (±20 °C).

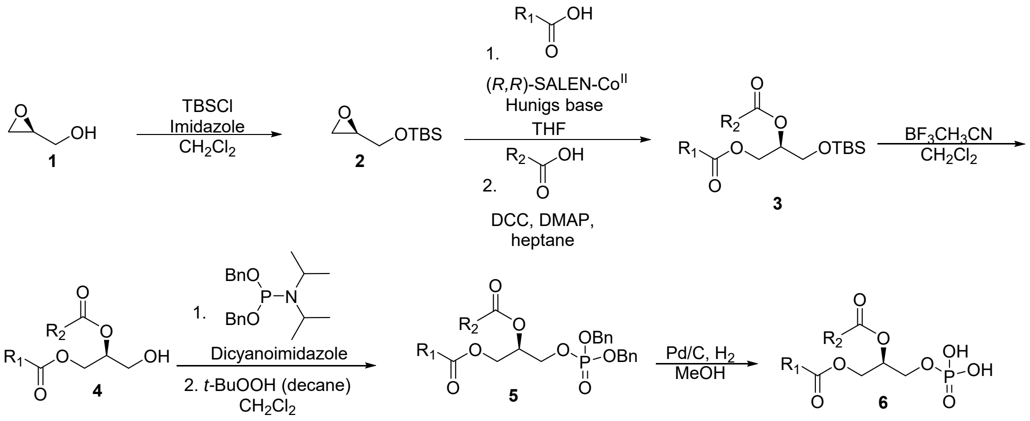

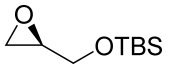

(*R*)-tert-butyldimethyl(oxiran-2-ylmethoxy)silane (**2**). To a stirred solution of imidazole (4.5 g, 66 mmol, 2.2 equiv) in CH_2Cl_2 (120 mL) was added TBSCl (5.0 g, 33 mmol, 1.1 equiv), resulting in a thick suspension that was cooled to 0 °C (ice/water bath). (*S*)-glycidol (2.2 g, 2 mL, 30 mmol) was added to in a dropwise fashion, after complete addition the mixture was allowed to warm up to room temperature and was stirred for 16 h. The suspension was filtered and washed with CH_2Cl_2, the filtrate was concentrated and further purified by column chromatography (5% Et_2O in pentane) giving the desired silyl ether was as a colourless oil (4.8 g, 25.5 mmol, 85%)

Spectral data correspond to previously reported[2]

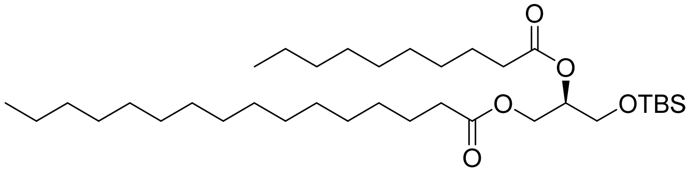

(*R*)-3-((tert-butyldimethylsilyl)oxy)-2-(decanoyloxy)propyl palmitate (**3a**)

To a deep red solution of *(R,R)*-SALEN-Co^{II} catalyst (12 mg, 0.020 mmol, 1 mol%) in Et_2O (2 mL) was added palmitic acid (513 mg, 2 mmol), immediately turning the mixture dark brown. The resulting solution was allowed to stir for 15 minutes before the solvent was evaporated. The dark brown residue was resolubilized by the addition of DIPEA (0.35 mL, 2 mmol, 1 equiv.) in THF (0.2 mL), to which epoxide **2** in THF (0.3 mL) was added. After 20 h, full conversion was observed via ^{1}H-NMR spectroscopy, whereupon the reaction mixture was concentrated. The resulting brown solid was dissolved in heptane (4 mL) to which decanoic acid (414 mg, 2.4 mmol, 1.2 equiv.) and DMAP (12 mg, 0.1 mmol, 5 mol%) were added. The resulting mixture was cooled to 0 °C (ice/water bath) and DCC (495 mg, 2.4 mmol, 1.2 equiv.) was added in one portion. After 16 h the entire mixture was transferred onto a flash-silica column and purified (2% Et_2O in pentane), yielding the protected diacylglycerol as a colourless thick liquid (980 mg, 1.64 mmol, 82%).

^{1}H NMR (400 MHz, Chloroform-*d*) δ 5.10 – 4.99 (m, 1H), 4.32 (dd, *J* = 11.8, 3.7 Hz, 1H), 4.13 (dd, *J* = 11.8, 6.3 Hz, 1H), 3.69 (dd, *J* = 5.4 Hz, 2H), 2.33 – 2.20 (m, 4H), 1.63 – 1.51 (m, 4H), 1.25 (br s, 36H), 0.88 – 0.82 (m, 15H), 0.03 (s, 6H).

^{13}C NMR (101 MHz, Chloroform-*d*) δ 173.4, 173.1, 71.8, 62.5, 61.6, 34.4, 34.2, 32.0, 32.0, 29.8, 29.8, 29.8, 29.7, 29.6, 29.5, 29.5, 29.4, 29.4, 29.2, 29.2, 25.8, 25.0, 25.0, 22.8, 22.8, 18.3, 14.2, 14.2, -5.4, -5.5.

HRMS-ESI$^+$ (*m/z*): $[M]^+$ calculated for $C_{35}H_{71}O_5Si$, 599.507; found, 599.507.

$[\alpha]_D^{20}$= +10.7 (c = 1 in $CHCl_3$).

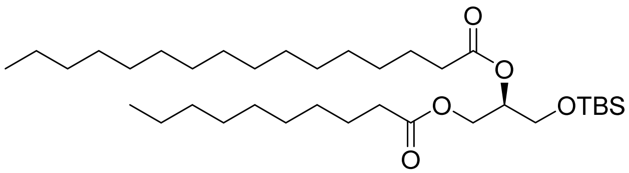

(*R*)-1-((tert-butyldimethylsilyl)oxy)-3-(decanoyloxy)propan-2-yl palmitate (**3b**) (380 mg, 2 mmol). This compound was prepared with the same synthetic procedure that was used for **(*R*)-3-((tert-butyldimethylsilyl)oxy)-2-(decanoyloxy)propyl palmitate** (**3a**) (980 mg, 1.64 mmol, 82%). Where decanoic acid was used in the cobalt mediated esterification, and palmitic acid in the Steglich esterification.

^{1}H NMR (400 MHz, Chloroform-*d*) δ 5.05 (m, 1H), 4.32 (dd, *J* = 11.9, 3.7 Hz, 1H), 4.14 (dd, *J* = 11.9, 6.3 Hz, 1H), 3.69 (dd, *J* = 5.3, 2H), 2.28 (m, 4H), 1.66 – 1.51 (m, 4H), 1.25 (br s, 36H), 0.98 – 0.73 (m, 15H), 0.03 (s, 6H).

^{13}C NMR (101 MHz, cdcl$_3$) δ 173.5, 173.5, 173.1, 173.1, 71.8, 62.5, 61.6, 34.4, 34.3, 32.0, 32.0, 29.8, 29.8, 29.8, 29.7, 29.6, 29.5, 29.5, 29.4, 29.4, 29.2, 29.2, 25.9, 25.0, 25.0, 22.8, 22.8, 18.3, 14.2, 14.2, -5.4, -5.4.

HRMS-ESI$^+$ (*m/z*): [M]$^+$ calculated for $C_{35}H_{71}O_5Si$, 599.507; found, 599.506

$[\alpha]_D^{20}$= +10.5 (c = 1 in $CHCl_3$).

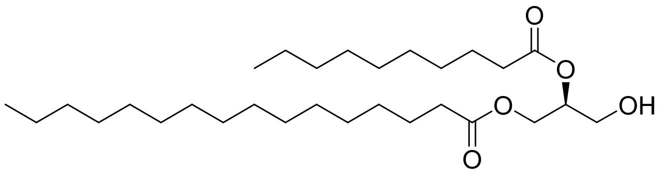

(S)-2-(decanoyloxy)-3-hydroxypropyl palmitate (**4a**). Silyl ether **3a** (180 mg, 0.3 mmol) was dissolved in CH_2Cl_2 and subsequently cooled to 0 °C (ice/water bath). $CH_3CN{\cdot}BF_3$ (0.275 mL, 0.33 mmol, 1.1 equiv) was added to the solution in a dropwise fashion and the mixture was allowed to stir for 5 minutes before it was diluted with 10 mL of Et_2O and poured onto ice cold phosphate buffer (1 M, pH = 7, 10 mL). The organic layer was extracted, washed with brine, dried over $MgSO_4$ and concentrated *in vacuo*, giving the free diacylglycerol as a white solid (145 mg, 0.3 mmol, 98%). Due to the instable nature of enantio-pure diacylglycerols the product was not further purified.

^{1}H NMR (400 MHz, Chloroform-*d*) δ 5.08 (p, *J* = 5.1 Hz, 1H), 4.31 (dd, *J* = 12.0, 4.4 Hz, 1H), 4.22 (dd, *J* = 12.0, 5.7 Hz, 1H), 3.72 (d, *J* = 5.1 Hz, 2H), 2.39 – 2.25 (m, 4H), 2.18 (br, 1H), 1.68 – 1.55 (m, 4H), 1.26 (br s, 36H), 0.87 (t, *J* = 6.6 Hz, 6H).

^{13}C NMR (101 MHz, cdcl$_3$) δ 173.9, 173.6, 72.3, 62.2, 61.7, 34.4, 34.3, 32.0, 32.0, 29.8, 29.8, 29.8, 29.8, 29.6, 29.6, 29.5, 29.4, 29.3, 29.2, 25.1, 25.0, 22.8, 22.8, 14.2, 14.2.

HRMS-ESI$^+$ (*m/z*): [M]$^+$ calculated for $C_{29}H_{57}O_5$, 485.420; found, 485.420

$[\alpha]_D^{20}$= -2.7 (c = 1 in $CHCl_3$).

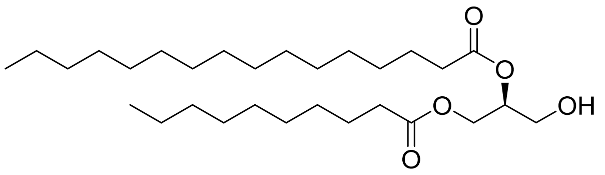

(S)-1-(decanoyloxy)-3-hydroxypropan-2-yl palmitate (**4b**) (280 mg, 0.39 mmol). This compound was prepared with the same synthetic procedure that was used for **(S)-2-(decanoyloxy)-3-hydroxypropyl palmitate** (**4a**) (165 mg, 0.34 mmol, 97%).

^{1}H NMR (400 MHz, Chloroform-*d*) δ 5.08 (p, *J* = 5.1 Hz, 1H), 4.31 (dd, *J* = 11.9, 4.4 Hz, 1H), 4.22 (dd, *J* = 11.9, 5.8 Hz, 1H), 3.71 (t, *J* = 5.1 Hz, 2H), 2.32 (dt, *J* = 8.9, 7.5 Hz, 4H), 2.21 (t, *J* = 6.1 Hz, 1H), 1.69 – 1.52 (m, 4H), 1.25 (br s, 36H), 0.86 (t, 6H).

^{13}C NMR (101 MHz, cdcl$_3$) δ 173.9, 173.6, 72.3, 62.2, 61.7, 34.4, 34.2, 32.0, 32.0, 29.8, 29.8, 29.8, 29.8, 29.6, 29.6, 29.5, 29.4, 29.4, 29.3, 29.2, 25.1, 25.0, 22.8, 22.8, 14.2, 14.2.

HRMS-ESI$^+$ (*m/z*): [M]$^+$ calculated for $C_{29}H_{57}O_5$, 485.420; found, 485.419

$[\alpha]_D^{20}$= -2.3 (c = 1 in CHCl$_3$).

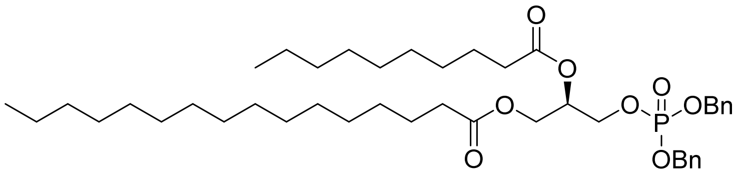

(R)-3-((bis(benzyloxy)phosphoryl)oxy)-2-(decanoyloxy)propyl palmitate (**5a**). Diacylglycerol **4a** (160 mg, 0.33 mmol) was dissolved in CH_2Cl_2 (1.3 mL), dibenzyl *N,N*-diisopropylphosphoramidite (0.145 mL, 0.43 mmol, 1.3 equiv) was added and the mixture was cooled to 0 °C (ice/water bath). 4,5-Dicyanoimidazole (47 mg, 0.4 mmol, 1.2 equiv) was added in one portion and the mixture was allowed to stir for 30 min before a 5 M solution of *t*-BuOOH (0.1 mL, 0.5 mmol, 1.5 equiv) was added in a dropwise fashion. After 30 minutes the mixture was diluted with ether and poured on a saturated $NaHCO_3$ solution. The layers were separated, and the organic layer was subsequently washed with 1 M HCl and brine, dried over $MgSO_4$ and concentrated *in vacuo*. The product was further purified by column chromatography (15% EtOAc in pentane) to afford the desired dibenzylphosphate **5a** (190 mg, 0.255 mmol, 77%) as a colourless oil with some minor co-eluting impurities.

^{1}H NMR (400 MHz, Chloroform-*d*) δ 7.41 – 7.29 (br s, 10H), 5.16 (p, *J* = 5.0 Hz), 5.09 – 4.95 (m, 4H), 4.25 (dd, *J* = 12.0, 4.4 Hz, 1H), 4.17 – 4.00 (m, 3H), 2.33 – 2.20 (m, 4H), 1.67 – 1.51 (m, 4H), 1.25 (br s, 36H), 0.87 (t, *J* = 6.9 Hz, 6H).

^{13}C NMR (101 MHz, cdcl$_3$) δ 173.2, 172.8, 135.8, 135.7, 135.7, 135.7, 128.7, 128.7, 128.0, 128.0, 69.6, 69.6, 69.4, 69.4, 65.5, 65.4, 61.7, 34.2, 34.1, 32.0, 32.0, 31.9, 29.8, 29.8, 29.7, 29.7, 29.6, 29.5, 29.4, 29.4, 29.2, 29.1, 24.9, 24.9, 22.8, 22.7, 14.2, 14.2.

^{31}P NMR (162 MHz, Chloroform-*d*) δ -1.03.

HRMS-ESI$^+$ (*m/z*): [M]$^+$ calculated for $C_{43}H_{70}O_8P$, 745.480; found, 745.470

$[\alpha]_D^{20}$= +2.4 (c = 1 in CHCl$_3$).

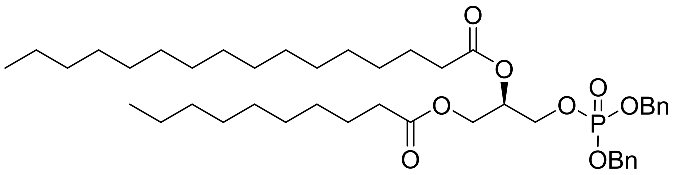

(R)-1-((bis(benzyloxy)phosphoryl)oxy)-3-(decanoyloxy)propan-2-yl palmitate (**5b**) (300 mg, 0.5 mmol). This compound was prepared with the same synthetic procedure that was used for **(R)-3-((bis(benzyloxy)phosphoryl)oxy)-2-(decanoyloxy)propyl palmitate** (**5a**) (165 mg, 0.22 mmol, 77%), which was also isolated with some minor co-eluting impurities.

^{1}H NMR (400 MHz, Chloroform-*d*) δ 7.42 – 7.29 (br s, 10H), 5.16 (p, *J* = 5.0 Hz, 1H), 5.09 – 4.94 (m, 4H), 4.25 (dd, *J* = 12.0, 4.4 Hz, 1H), 4.16 – 4.01 (m, 3H), 2.32 – 2.21 (m, 4H), 1.64 – 1.52 (m, 4H), 1.25 (br s, 36H), 0.88 (t, *J* = 6.8 Hz, 6H).

^{13}C NMR (101 MHz, Chloroform-*d*) δ 173.3, 172.9, 135.8, 135.8, 135.7, 135.7, 128.8, 128.8, 128.1, 128.1, 69.7, 69.6, 69.5, 69.4, 65.6, 65.5, 61.7, 34.3, 34.1, 32.1, 32.0, 29.8, 29.8, 29.8, 29.6, 29.6, 29.5, 29.4, 29.4, 29.2, 29.2, 24.8, 25.0, 22.8, 22.8, 14.2, 14.2.

^{31}P NMR (162 MHz, Chloroform-*d*) δ -1.03.

HRMS-ESI^{+} (*m/z*): [M]$^{+}$ calculated for $C_{43}H_{70}O_{8}P$, 745.480; found, 745.480

$[\alpha]_D^{20}$= +2.1 (c = 1 in $CHCl_3$).

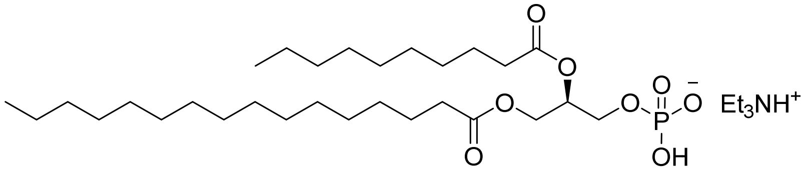

(*R*)-2-(decanoyloxy)-3-(palmitoyloxy)propyl hydrogen phosphate (**6a**). A solution of dibenzyl phosphate **5a** (52 mg, 70 µmol) in methanol (2 mL) was degassed with freeze-pump thaw technique. 10 w% Pd/C (4.3 mg, 4 µmol, 5 mol%) was added to the degassed mixture under nitrogen atmosphere. Subsequently a hydrogen atmosphere was established, and the suspension was allowed to stir for 16 h. The black mixture was diluted with Et_2O and filtered over celite, the filtrate was concentrated and further purified by column chromatography (20% MeOH and 1% Et_3N in $CHCl_3$). The desired Et_3NH^+ phosphate salt **6a** (28 mg, 50 µmol, 71%) was isolated as an oil. A general remark for this procedure; all impurities were stained with PMA while the product could be stained with bromocresol green.

^{1}H NMR (400 MHz, Chloroform-*d*) δ 8.82 (s, 1H), 5.22 (s, 1H), 4.38 (dd, *J* = 12.1, 3.1 Hz, 1H), 4.17 (dd, *J* = 12.1, 7.0 Hz, 1H), 4.00 (t, *J* = 6.2 Hz, 2H), 3.06 (s, 6H), 2.27 (q, *J* = 7.5 Hz, 4H), 1.65 – 1.51 (m, 4H), 1.24 (br s, 45H), 0.87 (t, *J* = 6.7 Hz, 6H).

^{13}C NMR (101 MHz, cdcl$_3$) δ 173.5, 173.2, 70.6, 70.5, 63.5, 63.0, 45.7, 34.4, 34.2, 32.1, 32.0, 29.8, 29.8, 29.8, 29.7, 29.6, 29.5, 29.5, 29.3, 29.3, 25.1, 25.0, 22.8, 22.8, 14.2, 8.7.

^{31}P NMR (162 MHz, Chloroform-*d*) δ 1.61.

HRMS-ESI$^-$ (*m/z*): [M]$^-$ calculated for $C_{29}H_{56}O_8P$, 563.372; found, 563.372

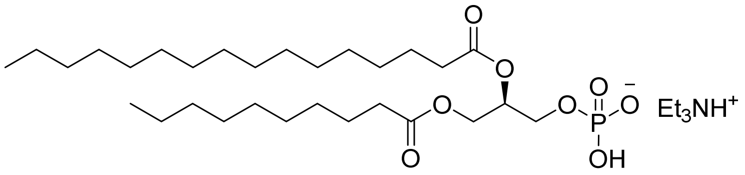

(R)-3-(decanoyloxy)-2-(palmitoyloxy)propyl hydrogen phosphate (**6b**) (45 mg, 60 µmol). This compound was prepared with the same synthetic procedure that was used for **(*R*)-2-(decanoyloxy)-3-(palmitoyloxy)propyl hydrogen phosphate** (**6a**) (26 mg, 46 µmol, 76%).

^{1}H NMR (400 MHz, Chloroform-*d*) δ 5.22 (s, 1H), 4.37 (d, *J* = 11.9 Hz, 1H), 4.16 (dd, *J* = 12.0, 6.7 Hz, 1H), 4.08 – 3.85 (m, 2H), 3.09 (s, 6H), 2.27 (q, *J* = 7.4 Hz, 4H), 1.65 – 1.50 (m, 5H), 1.26 (br s, 45H), 0.87 (t, *J* = 6.6 Hz, 6H).

^{13}C NMR (101 MHz, Chloroform-*d*) δ 173.5, 173.2, 70.5, 65.5, 62.9, 45.8, 34.4, 34.2, 32.06, 32.0, 29.7, 29.8, 29.8, 29.7, 29.7, 29.6, 29.5, 29.5, 29.5, 29.4, 29.3, 25.1, 25.0, 22.8, 22.8, 14.2, 8.7.

^{31}P NMR (162 MHz, Chloroform-*d*) δ 1.54.

HRMS-ESI$^-$ (*m/z*): [M]$^-$ calculated for $C_{29}H_{56}O_8P$, 563.372; found, 563.372

Supplementary References

---

[1] Armarego, W. L., & Chai, C. L. L. *Purification of laboratory chemicals* (Butterworth-Heinemann, 2013).

[2] Fodran, P. & Minnaard, A. J. Catalytic synthesis of enantiopure mixed diacylglycerols – synthesis of a major M. tuberculosis phospholipid and platelet activating factor. *Org. Biomol. Chem.* **11**, 6919 (2013).

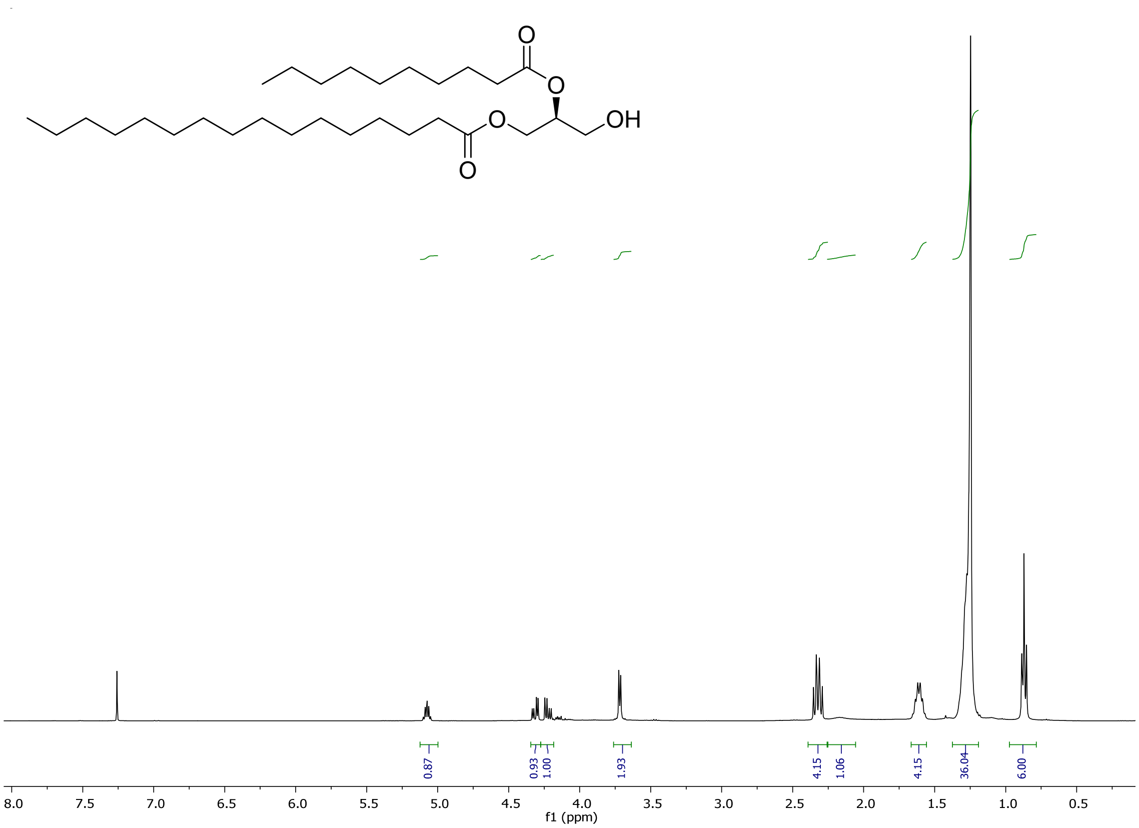

Supplementary figure 11: ^{1}H NMR spectra for **4a**

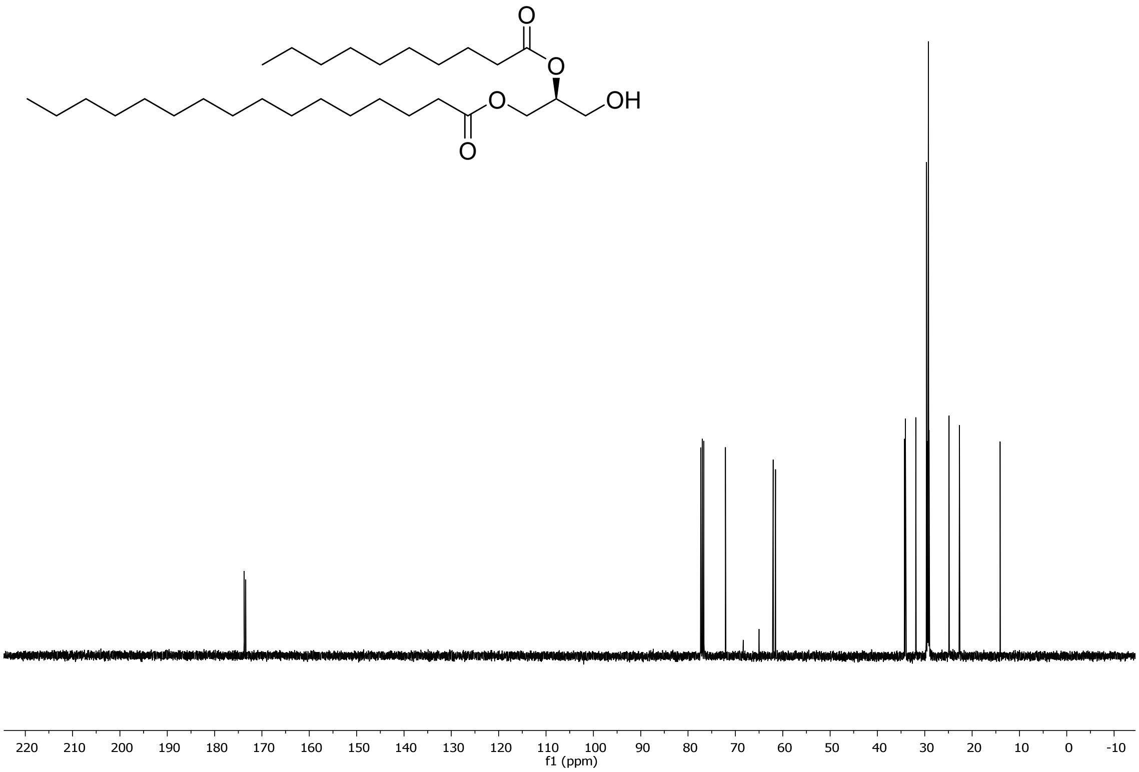

Supplementary figure 12: ^{13}C NMR spectra for **4a**

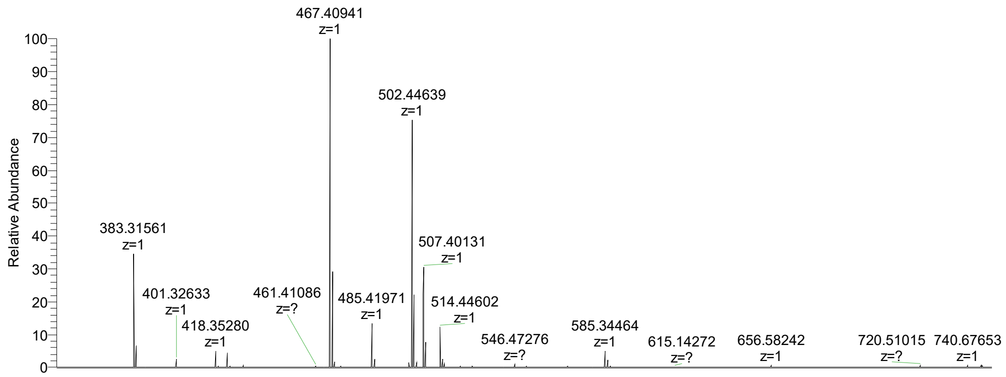

Supplementary figure 13: HRMS spectra for **4a**

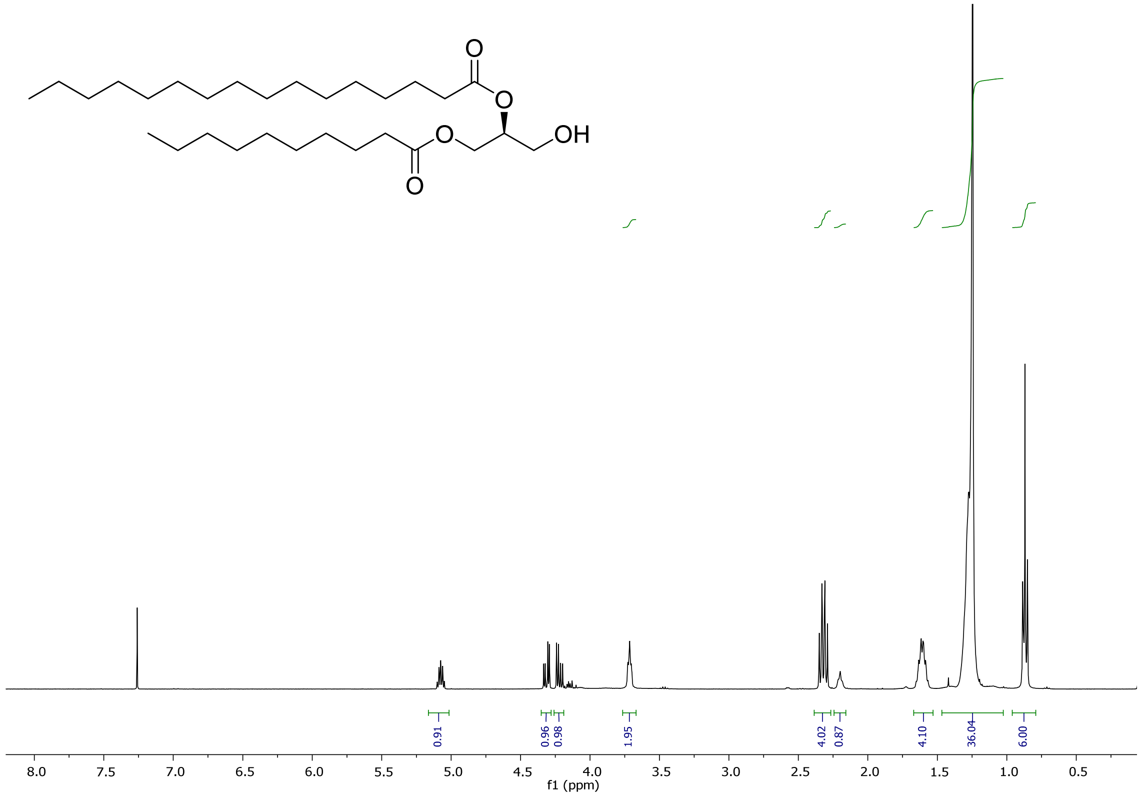

Supplementary figure 14: ^{1}H NMR spectra for **4b**

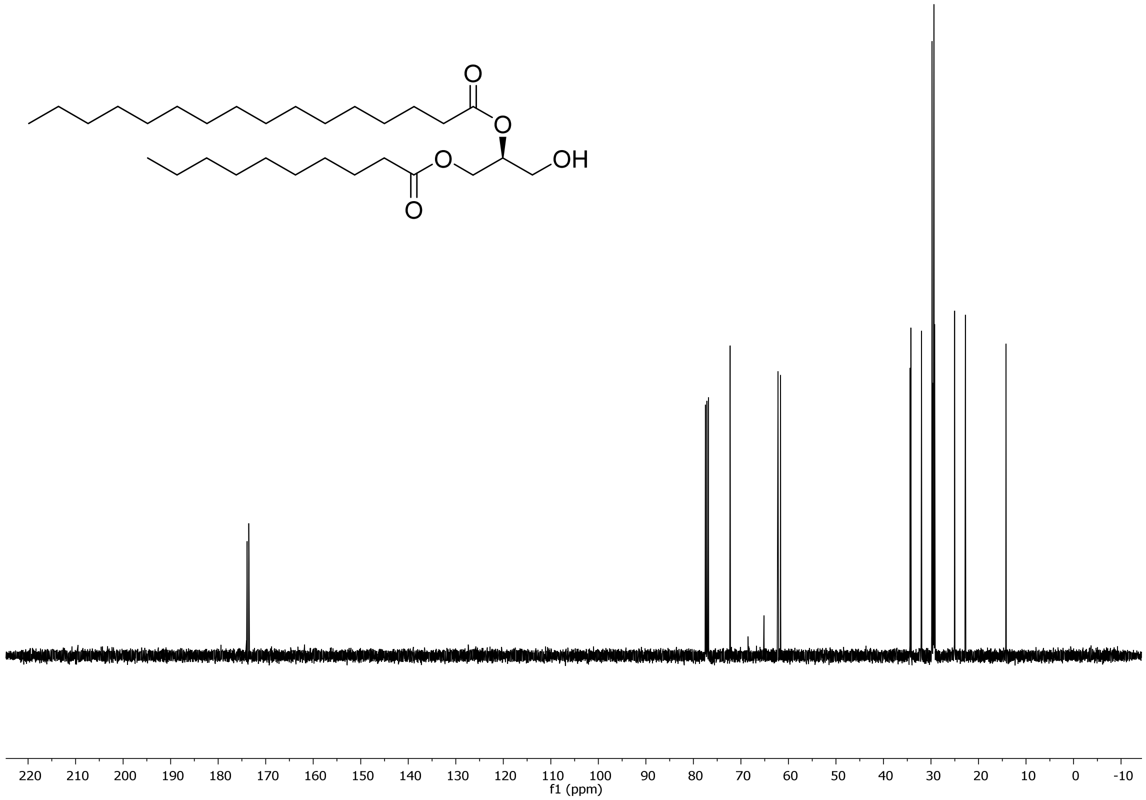

Supplementary figure 15: ^{13}C NMR spectra for **4b**

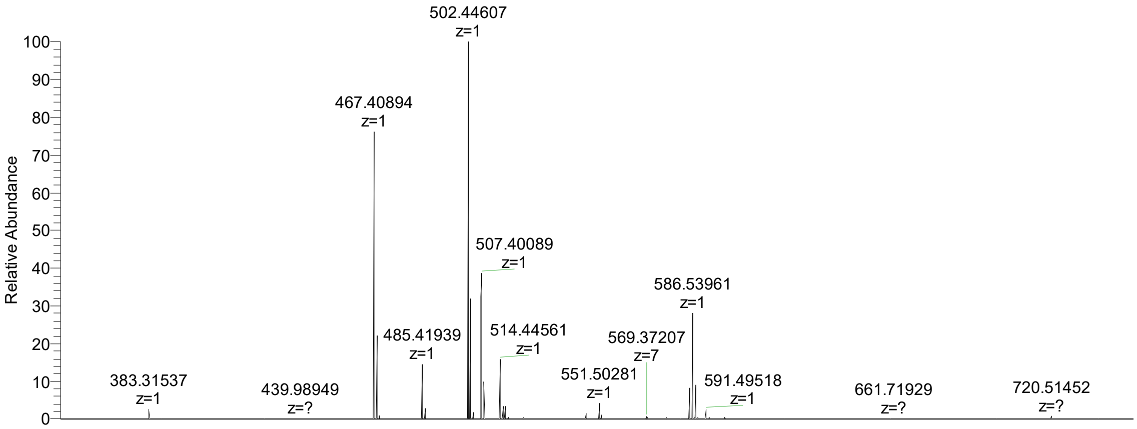

Supplementary figure 16: HRMS spectra for **4b**

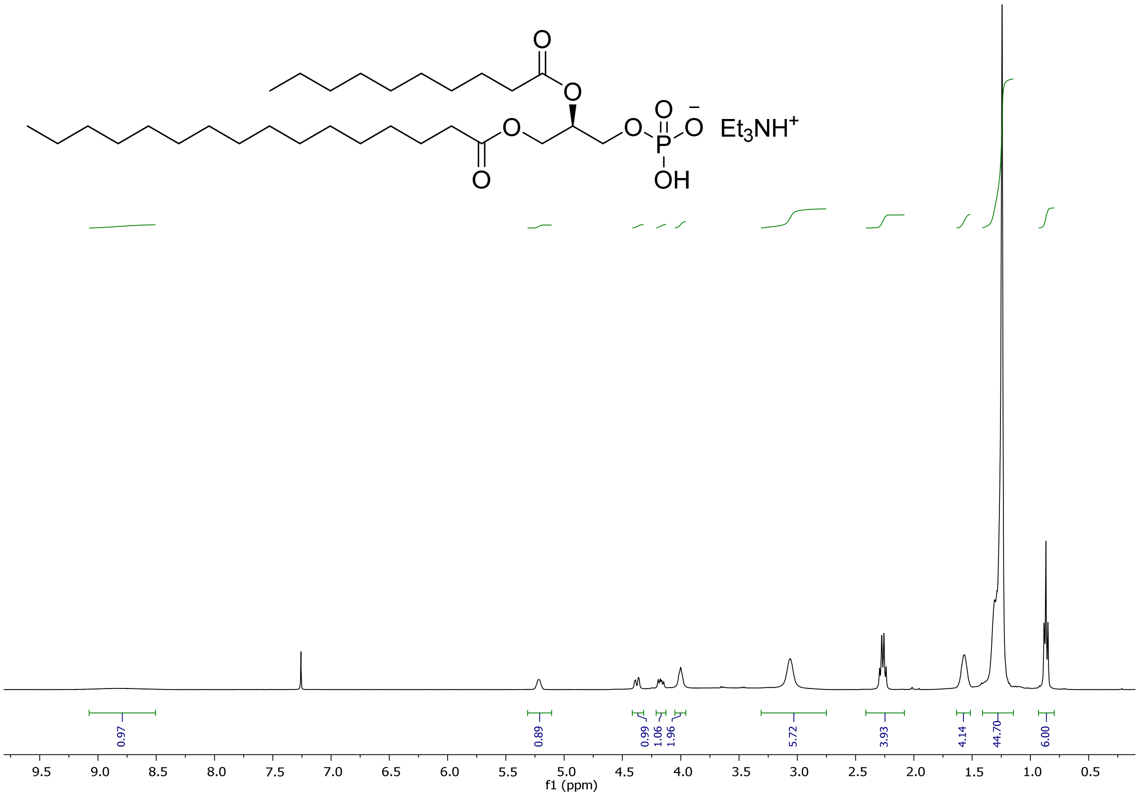

Supplementary figure 17: ^{1}H NMR spectra for **6a**

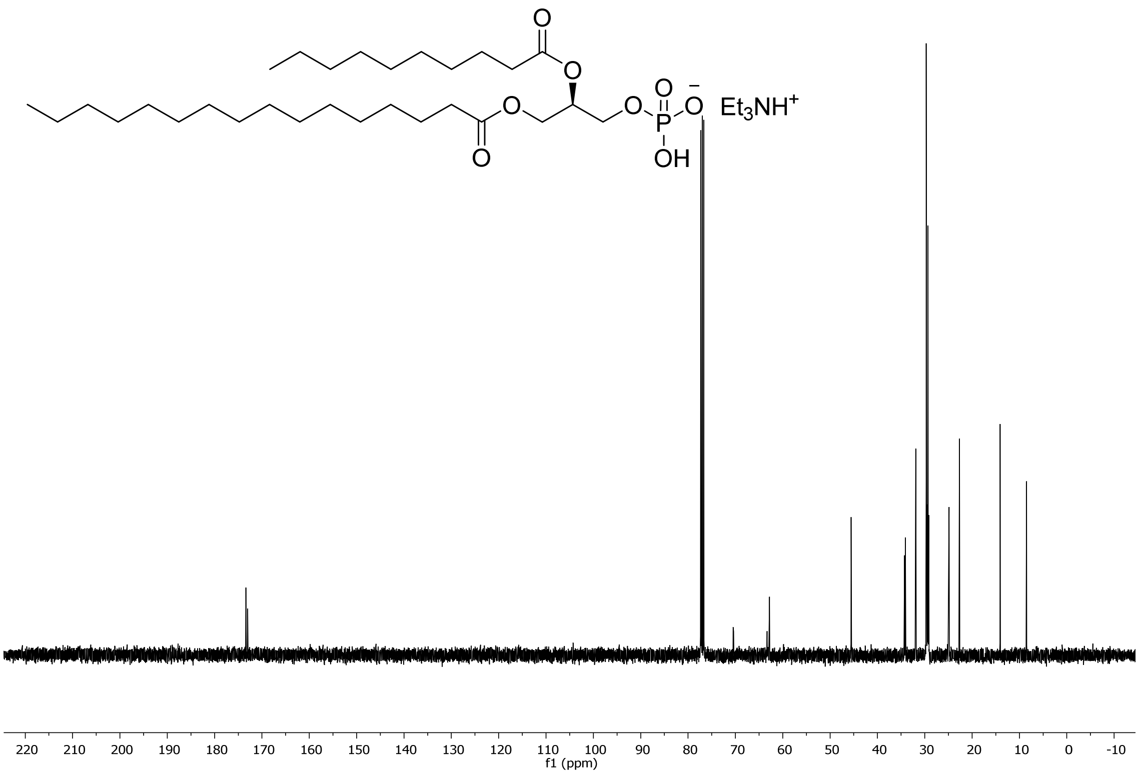

Supplementary figure 18: ^{13}C NMR spectra for **6a**

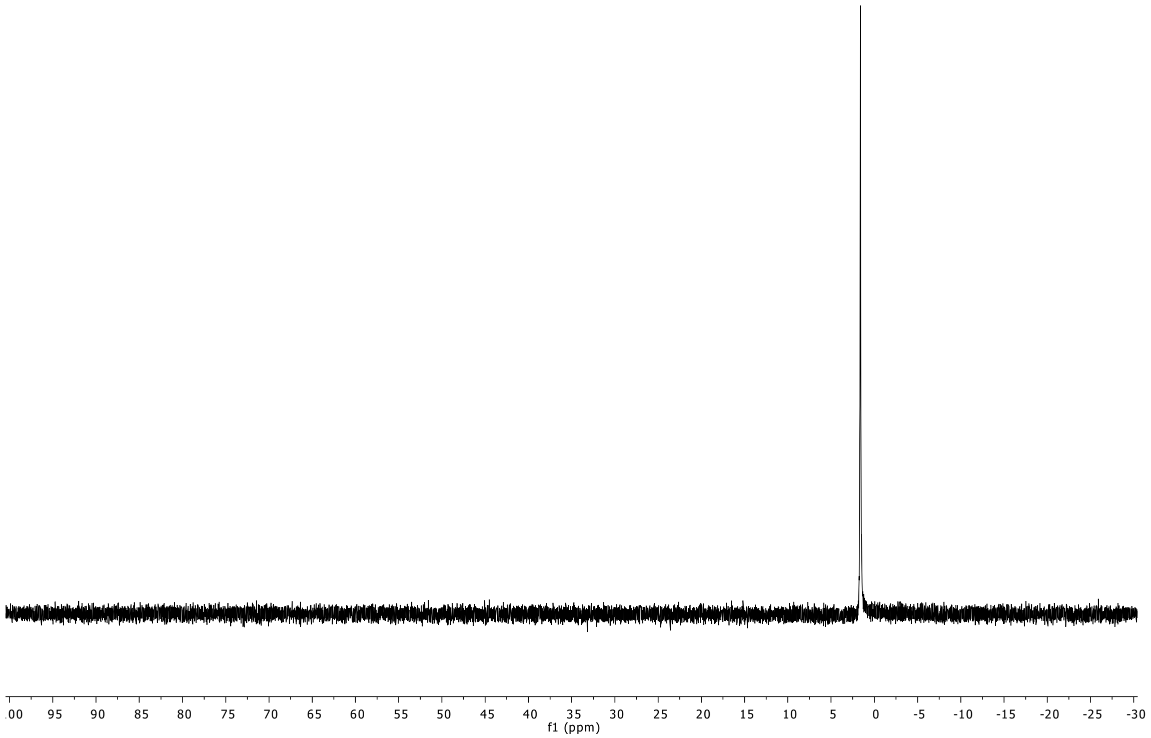

Supplementary figure 19: ^{31}P NMR spectra for **6a**

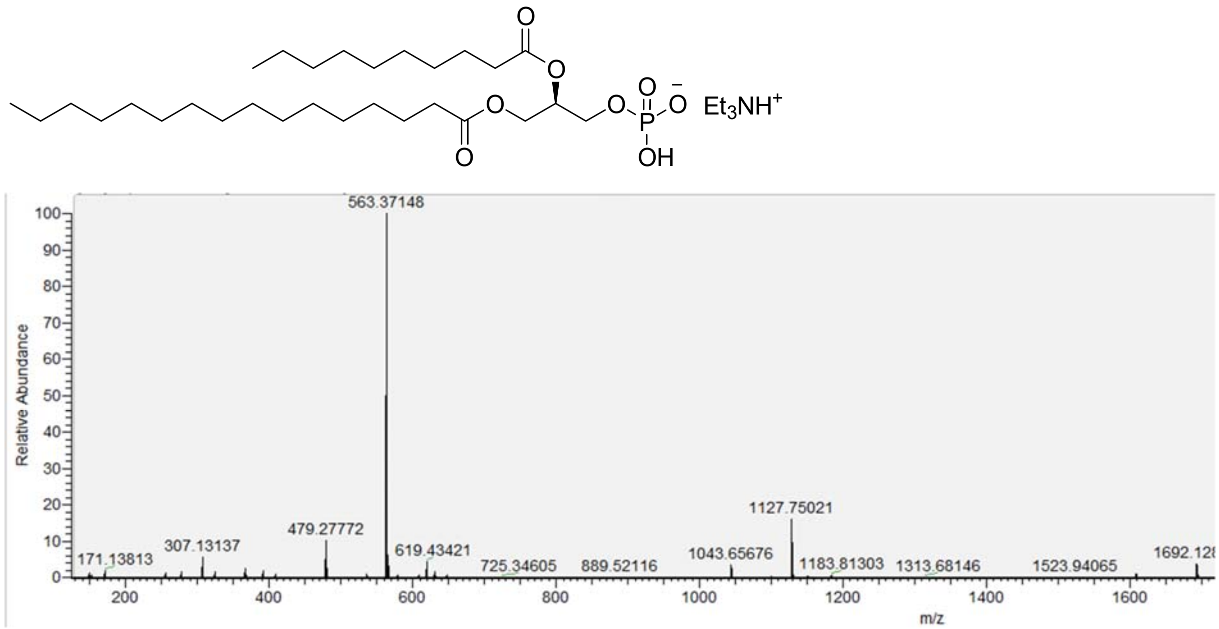

Supplementary figure 20: HRMS spectra for **6a**

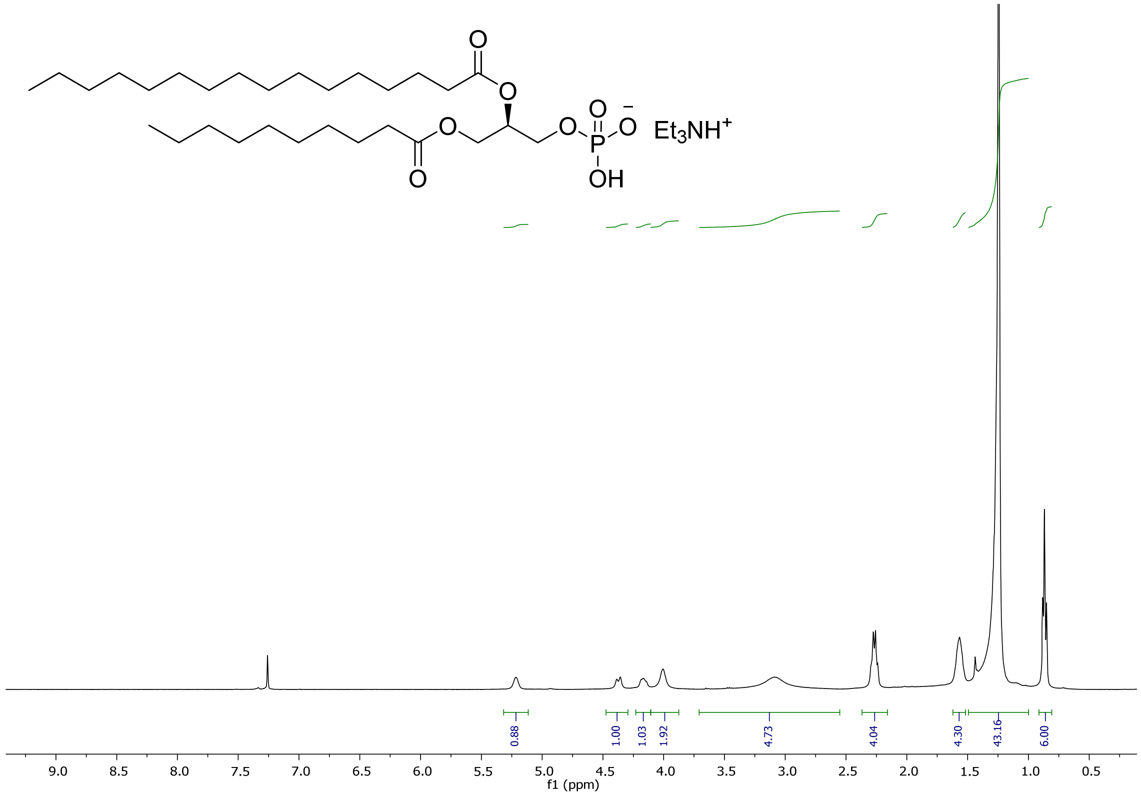

Supplementary figure 21: ^{1}H NMR spectra for **6b**

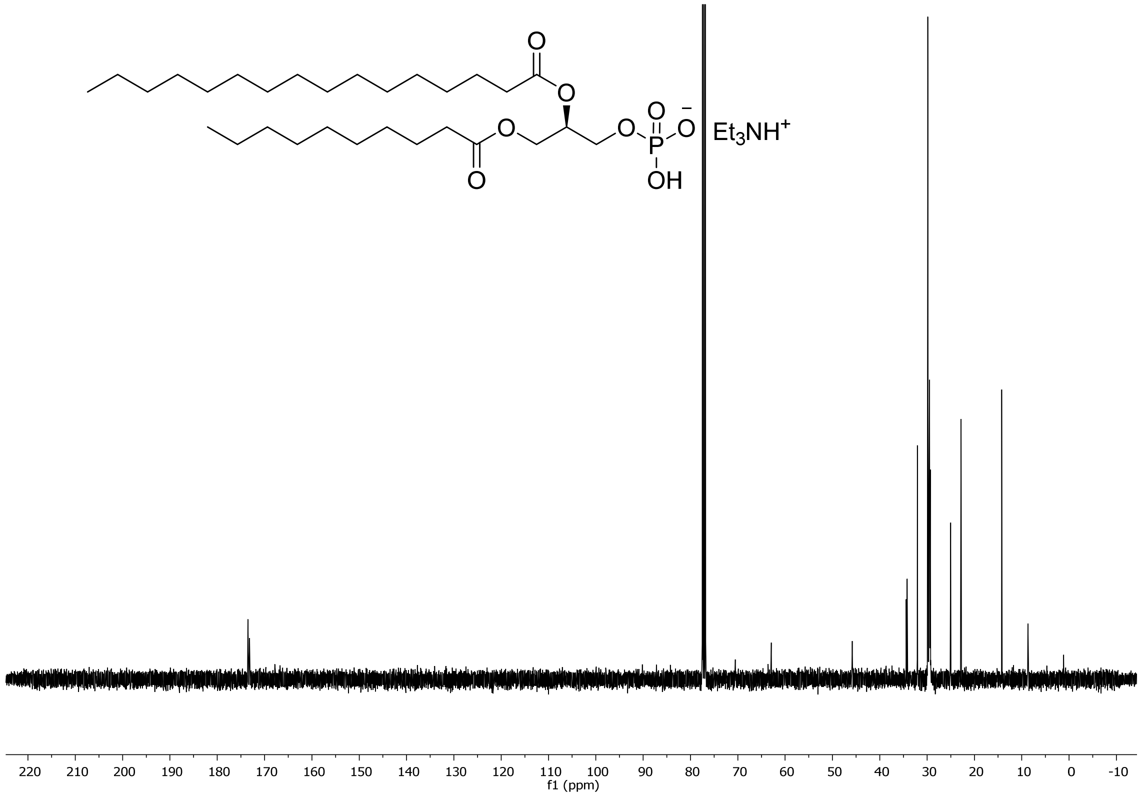

Supplementary figure 22: ^{13}C NMR spectra for **6b**

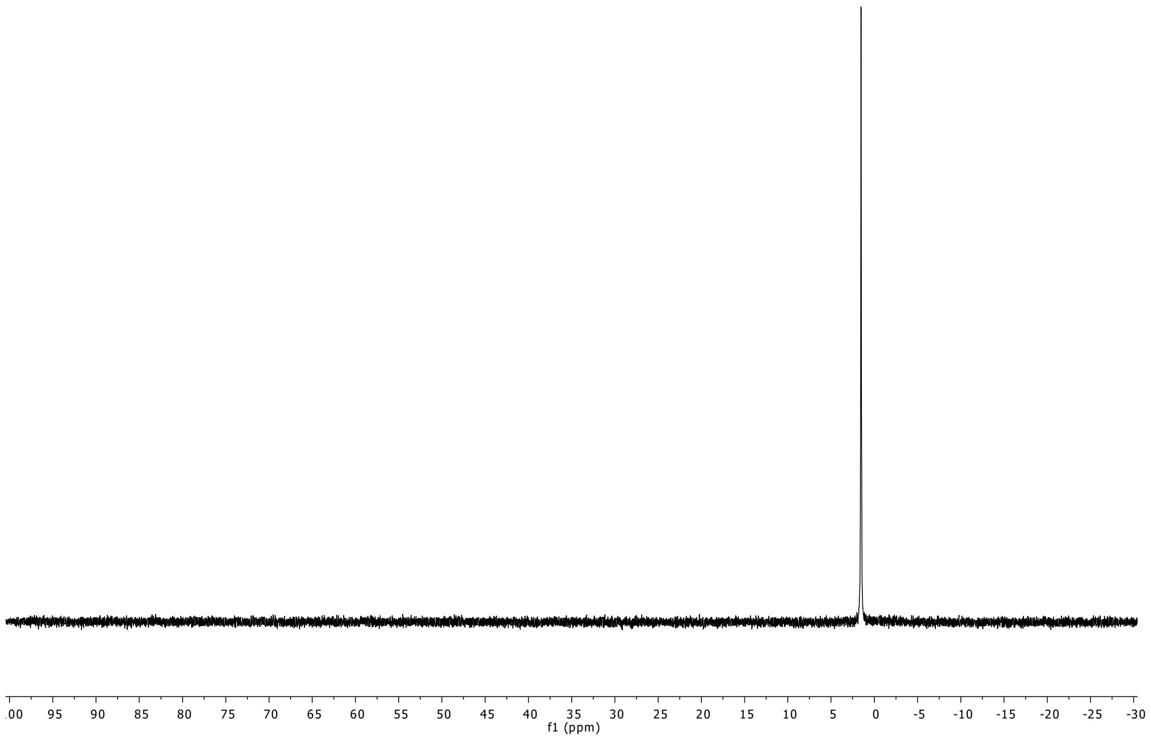

Supplementary figure 23: ^{31}P NMR spectra for **6b**

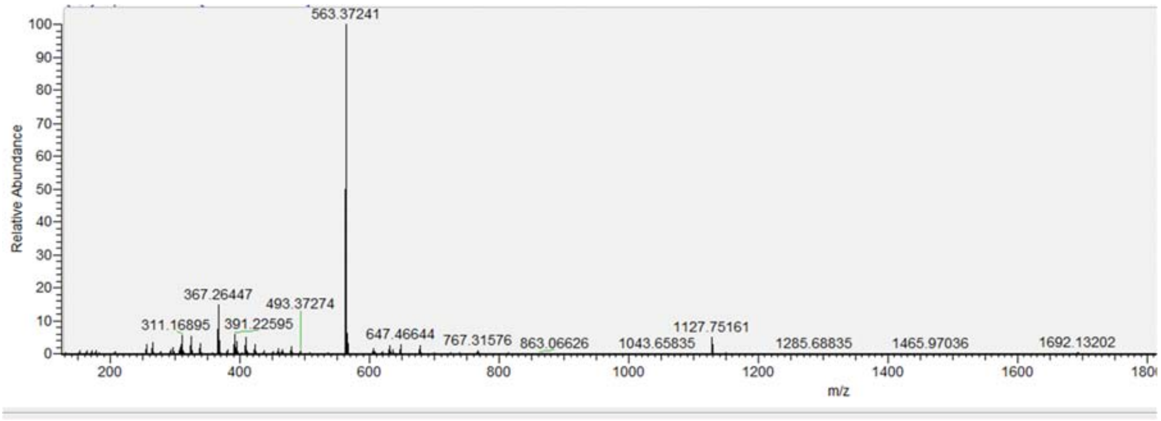

Supplementary figure 24: HRMS spectra for **6b**

-
