# Supplementary figures and images for: The late stage of COPI vesicle fission requires shorter forms of phosphatidic acid and diacylglycerol
Source: Nat Commun. 2019 Jul 30;10:3409. doi: 10.1038/s41467-019-11324-4 (PMC6667475; doi:10.1038/s41467-019-11324-4)

## Slide 1
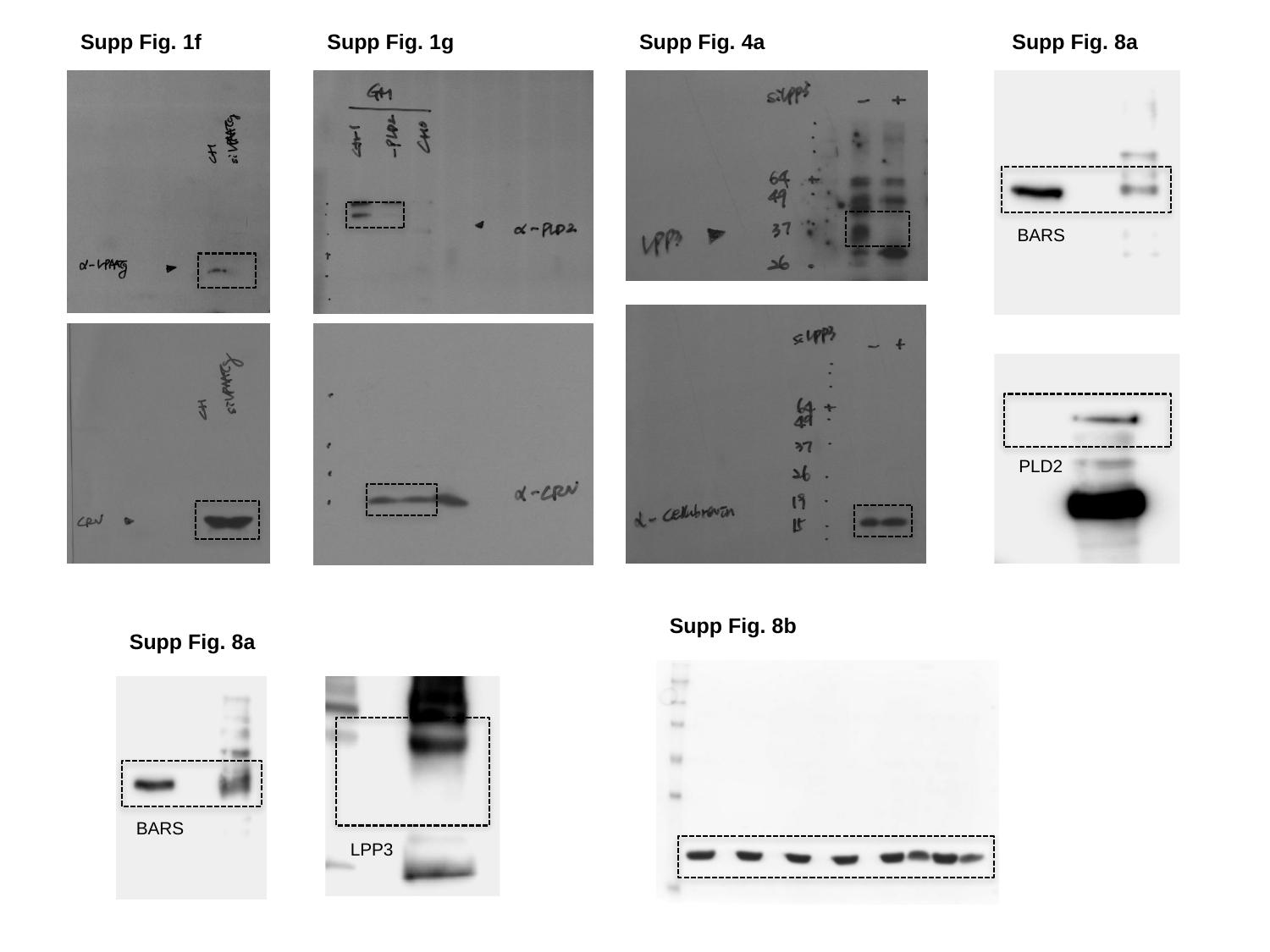

Supp Fig. 1f
Supp Fig. 1g
Supp Fig. 4a
Supp Fig. 8a
BARS
PLD2
Supp Fig. 8b
Supp Fig. 8a
BARS
LPP3

Supplement: Supplementary file 3 — Source Data [file 41467_2019_11324_MOESM3_ESM.zip › Source data.rev/Gel scans.pptx]
